# Multiscale study of mononuclear Co^II^ SMMs based on curcuminoid ligands†
†Electronic supplementary information (ESI) available: Detailed description of materials and methods, crystallography, physical methods, computational methods, paramagnetic ^1^H NMR, UV-vis, AFM and photoemission. CCDC 1411573 (**1**) and 1411574 (**2**). For ESI and crystallographic data in CIF or other electronic format see DOI: 10.1039/c5sc03298a


**DOI:** 10.1039/c5sc03298a

**Published:** 2016-01-07

**Authors:** Raúl Díaz-Torres, Melita Menelaou, Olivier Roubeau, Alessandro Sorrenti, Guillem Brandariz-de-Pedro, E. Carolina Sañudo, Simon J. Teat, Jordi Fraxedas, Eliseo Ruiz, Núria Aliaga-Alcalde

**Affiliations:** a Departament de Química Inorgànica , Universitat de Barcelona , Diagonal 645 , 08028 Barcelona , Spain; b Instituto de Ciencia de Materiales de Aragón (ICMA) , CSIC and Universidad de Zaragoza , Plaza San Francisco s/n , 50009 Zaragoza , Spain; c CSIC-ICMAB (Institut de Ciència dels Materials de Barcelona) Campus de la Universitat Autònoma de Barcelona , 08193 Bellaterra , Spain; d Advanced Light Source , Lawrence Berkeley National Laboratory , Berkeley , California 94720 , USA; e Catalan Institute of Nanoscience and Nanotechnology (ICN2), CSIC and The Barcelona Institute of Science and Technology , Campus UAB, Bellaterra , 08193 Barcelona , Spain; f Institut de Química Teòrica i Computacional , Universitat de Barcelona , Diagonal, 645 , 08028 Barcelona , Spain; g ICREA (Institució Catalana de Recerca i Estudis Avançats) , CSIC-ICMAB (Institut de Ciència dels Materials de Barcelona) Campus de la Universitat Autònoma de Barcelona , 08193 Bellaterra , Spain . Email: nuria.aliaga@icrea.cat; h Departament de Química Inorgànica and Institut de Nanociència i Nanotecnologia-UB , Universitat de Barcelona , Diagonal 645 , 08028 Barcelona , Spain

## Abstract

This work introduces a novel family of Co^II^ species having a curcuminoid (CCMoid) ligand, 9Accm, attached, namely [Co(9Accm)_2_(py)_2_] (**1**) and [Co(9Accm)_2_(2,2′-bpy)] (**2**), achieved in high yields by the use of a microwave reactor, and exhibiting two different arrangements for the 9Accm ligands, described as “*cis*”(**2**) and “*trans*”(**1**). The study of the similarities/differences of the magnetic, luminescent and surface behaviors of the two new species, **1** and **2**, is the main objective of the present work. The determined single-crystal structures of both compounds are the only Co^II^-CCMoid structures described in the literature so far. Both compounds exhibit large positive *D* values, that of **1** (*D* = +74 cm^–1^) being three times larger than that of **2** (*D* = +24 cm^–1^), and behave as mononuclear Single-Molecule Magnets (SMMs) in the presence of an external magnetic field. Their similar structures but different anisotropy and SMM characteristics provide, for the first time, deep insight on the spin-orbital effects thanks to the use of CASSCF/NEVPT2 calculations implementing such contributions. Further magnetic studies were performed in solution by means of paramagnetic ^1^H NMR, where both compounds (**1** and **2**) are stable in CDCl_3_ and display high symmetry. Paramagnetic NMR appears to be a useful diagnostic tool for the identification of such molecules in solution, where the resonance values found for the methine group (–CH–) of 9Accm vary significantly depending on the *cis* or *trans* disposition of the ligands. Fluorescence studies show that both systems display chelation enhancement of quenching (CHEQ) with regard to the free ligand, while **1** and **2** display similar quantum yields. Deposition of **1–2** on HOPG and Si(100) surfaces using spin-coating was studied using AFM; UV photoemission experiments under the same conditions display **2** as the most robust system. The measured occupied density of states of **2** with UV photoemission is in excellent agreement with theoretical DFT calculations.

## Abstract 2

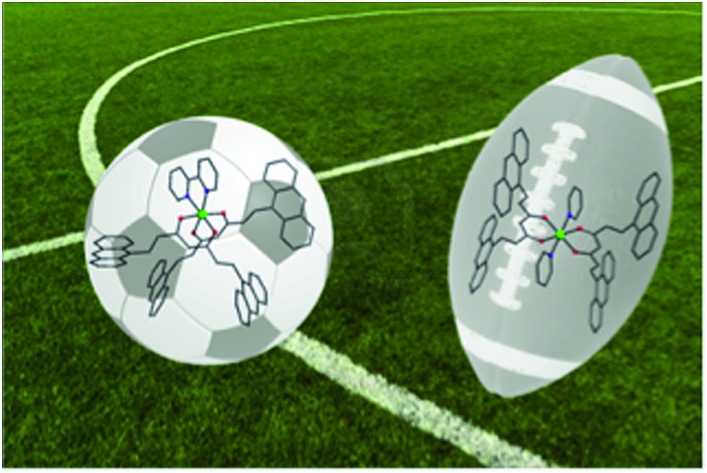
Two related single-molecule magnets, [Co(9Accm)_2_(py)_2_] and [Co(9Accm)_2_(2,2′-bpy)], with *cis* and *trans* disposition of the CCMoid ligands were studied in solution, in the solid state and by deposition on different surfaces.

## Introduction

Within a variety of frameworks and different time periods, fields like molecular electronics,^1^ molecular magnetism1a,^2^ and molecular spintronics2a–c,^3^ have pointed out the relevance of organic systems and coordination compounds toward their application in nanoscience and nanotechnology.^1^–^4^ For that, the reliable characterization of the performance of such entities not only in the solid state and solution but also on surfaces/devices is mandatory.^5^

Small and rather straightforward coordination compounds provide effective solutions allowing clear understanding of their functioning and improving fundamental and applied research. Great efforts are directed toward the design of molecular compounds taking into account the overall effects of the organic ligands and metals attached,^6^ as this task is not always easy to anticipate. In the metal–ligand consortium, the former can provide redox, magnetic and/or luminescent properties,^2^,^7^ among others, and tune others like optical performance^8^ or luminescence.^9^ This together with the power of organic matter to introduce new properties allows suitable functional materials to be created by the synergy of both. In this sense, mononuclear coordination compounds are gaining relevance as autonomous units that ultimately can function as building blocks^10^ in more elaborated structures.

To facilitate the correct development of the above mentioned fields, further insight into the factors that affect the final properties is crucial, including stability and robustness. Realistic use of molecular materials also implies the study of performance upon deposition on surfaces^11^ and among electrodes/inside devices.1a,^12^

**Scheme 1:**
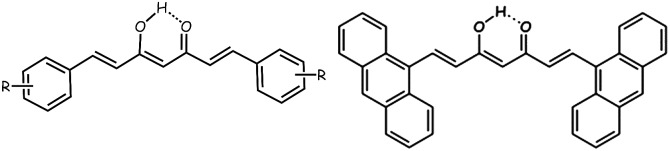
A general drawing of a symmetric CCMoid (left) and the ligand 9Accm (right) in their enol forms.

We direct our efforts to integrate mononuclear functional coordination compounds into the areas described above, by giving relevance to both the metal center and the organic ligands attached to it. The organic groups selected for such an enterprise are curcumin derivatives also called curcuminoids (CCMoids), depicted in Scheme 1, left. CCMoids are synthetic bio-inspired molecules well-known in bio-oriented fields^13^ and recently introduced in molecular magnetism and molecular electronics by some of us.^14^–^16^ In particular, the ligand used in this work, 9Accm (Scheme 1, right),^14^ was tested at the nanoscale, behaving as a nanowire capable of electronic transport in carbon-based gateable molecular junctions.^15^ Attached to metals, 9Accm has produced complexes with relevant biological, magnetic or visible/near-IR luminescent properties.^9^,^16^ Apart from its fluorescent properties, 9Accm appears to be an excellent platform to contact graphene electrodes or to attach coordination compounds on carbon-based surfaces.^15^ We are interested in the study of such affinity applied now to a family of cobalt coordination compounds.

Here, we introduce two novel hexacoordinated Co^II^ compounds, [Co(9Accm)_2_(py)_2_] (**1**) and [Co(9Accm)_2_(2,2′-bpy)] (**2**), which are to the best of our knowledge the only two systems crystallographically described using CCMoid ligands. Compounds **1** and **2** differ in the disposition of the coordinated 9Accm ligands. The present work aims to relate the magnetic/fluorescent responses of two Co^II^ compounds with the inherent properties that the arrangement of the ligands confers to the final compounds in the bulk, in solution and on surfaces. Studies in the solid state show that **1** and **2** present almost identical ligands, and do not exhibit highly-distorted coordination environments but clearly differ magnetically due to the tuning of the metal coordination. Insight into spin-orbital effects has been accomplished through theoretical calculations. This thorough analysis includes a comparison with the limited family of mononuclear Co^II^ hexacoordinated SMMs. Studies of the stability of the two systems in solution were targeted by the use of a paramagnetic ^1^H NMR technique with subsequent fluorescence experiments. To describe the affinity and robustness, the deposition processes of **1** and **2** on HOPG/Si(100) substrates are described together with their analyses using photoemission experiments, corroborated by theoretical studies as well.

## Experimental

Synthesis of [Co(9Accm)_2_(py)_2_] (**1**). The new system was synthesized by adding 26 mg of [Co(O_2_CMe)_2_·4H_2_O] (0.104 mmol) together with 100 mg of 9Accm (0.210 mmol) in 5 mL of pyridine into a microwave (MW) tube and most of the free ligand remained insoluble. The MW conditions allowed the temperature and pressure to rise freely at the same time as strong stirring was applied. After less than 2 min the maximum temperature was reached (140 °C) and was kept constant for another 2 min. The reaction was then cooled to room temperature, resulting in a clear brown solution from which nice crystals were directly isolated after several hours of standing. Yield: 102 mg (83%). Anal. calcd for C_80_H_56_CoN_2_O_4_·0.2C_5_H_5_N (1183.17 g mol^–1^): C 82.16; H 4.85; N 2.60. Found: C 82.06; H 4.73; N 2.50. IR data (KBr, cm^–1^): 3434(br), 3048(w), 3016(w), 2925(w), 2846(w), 1632(w), 1558(m), 1504(s), 1441(s), 1349(w), 1296(w), 1259 (w), 1212(w) 1162(w), 970(w), 887(w), 734(m), 696(w), 444(w). MALDI^+^ (DHB) (*m*/*z*): 1010.3 ([Co(9Accm)_2_ + H]^+^ and 1032.3 ([Co(9Accm)_2_ + Na]^+^).

Synthesis of [Co(9Accm)_2_(bpy)] (**2**). Compound **2** was obtained using identical MW parameters as before, by adding 26 mg of [Co(O_2_CMe)_2_·4H_2_O] (0.104 mmol), 100 mg of 9Accm (0.210 mmol) and 16 mg of 2,2′-bipyridine (0.102 mmol) to a MW tube using 5 mL of DMF as the solvent. Yield: 107 mg (88%). Crystals suitable for analyses were achieved by slow evaporation of a CHCl_3_ solution of the final solid. Anal. calcd for C_80_H_54_CoN_2_O_4_·0.2C_3_H_7_NO (1179.95 g mol^–1^): C 81.98; H 4.73; N 2.61. Found: C 81.83; H 4.63; N 2.48. IR data (KBr, cm^–1^): 3429(br), 3043(w), 3021(w), 2994(w), 2917(w), 2848(w), 2087(w), 1672(m), 1630(m), 1598(w), 1551(m), 1506(s),1442(m), 1351(m), 1311(w), 1264(w), 1161(m), 1017(w), 968(m), 879(m), 842(w), 763(m), 733(s), 602(w), 540(w), 446(w). MALDI^+^ (DHB) (*m*/*z*): 690.1 ([Co(9Accm)(2,2′-bpy)]^+^) and 1032.3 ([Co(9Accm)_2_ + Na]^+^).

## Results and discussion

### Synthesis

Compound **1** [Co(9Accm)_2_(py)_2_], and **2**, [Co(9Accm)_2_(2,2′-bpy)], were synthesized using a microwave (MW) reactor. This methodology, well-established for organic molecules,^17^ has also been described in the past for the achievement of coordination compounds^17^ and used by some of us in related compounds to those described here.16*a* In our experience, a microwave assisted technique has improved yields and allowed the amount of starting materials used to be increased, drastically decreasing the volume of the required solvents together with reaction times.16a,^17^,^18^ In the case of pyridine (compound **1**), crystals were obtained directly from the microwave tube after cooling down the reaction. Here, the presence of pyridine or 2,2′-bipyridine is the key factor for the reorganization of 9Accm around the Co^II^ centers and is therefore responsible for the differences between **1** and **2**.

### Structural descriptions

Compounds **1** and **2** are the first Co-CCMoids crystallographically described in the literature so far.

**Fig. 1:**
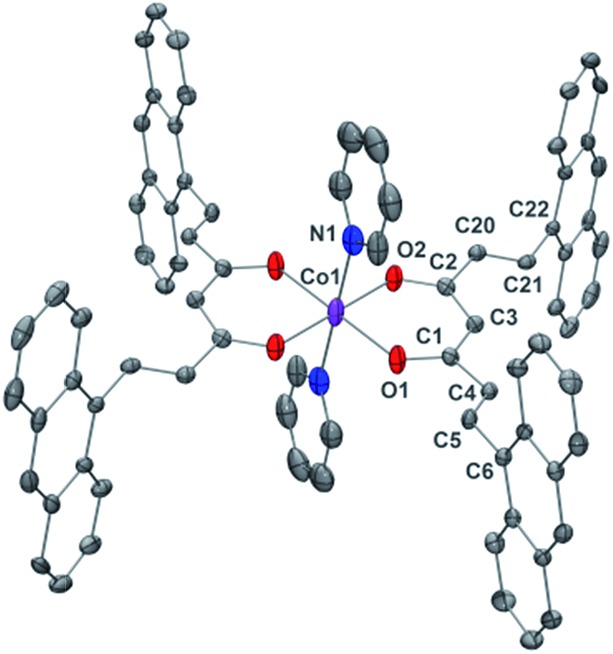
POV-Ray view of **1** with thermal ellipsoids fixed at 30%. Protons are omitted for the sake of simplification. Color legend: Co in magenta, O in red, N in blue and C in grey.

General crystal data information of the two species is presented in Table S1.† Compound **1**, [Co(9Accm)(py)_2_], crystallizes in the monoclinic space group *P*2_1_/*c*. The mononuclear species contain one hexacoordinated Co^II^ centre that binds two molecules of 9Accm and two molecules of pyridine. The organic pairs of ligands display a *trans* conformation providing a *D*_4h_ ideal geometry. Selected bond lengths and angles are listed in Table S1† and Fig. 1 shows a POV-Ray projection for compound **1**. This molecule shows two Co–O distances of 2.002(2) and 2.033(2) Å and one Co–N distance of 2.209(4) Å, in agreement with others reported elsewhere.^19^ The O(1)–Co–O(2′) and O(1)–Co–O(2) angles are 89.88 and 90.13°, respectively, while O(1)–Co–O(1′), O(2)–Co–O(2′) and N(1)–Co–N(1′) are all 180° by symmetry. Basically, the coordinated 9Accm ligands display two alternating C–C values: C(1)–C(4), C(5)–C(6), C(2)–C(20) and C(21)–C(22) relate to single C–C distances (1.400–1.486 Å) and on the other hand, C(4)–C(5) and C(20)–C(21) show characteristic double C–C bonds (between 1.311 and 1.315 Å). Such distances are found in related compounds.^14^,^16^ It must be stressed that the conjugated chains in the two sides of the ligand have a different conformation, either zig-zag or boat shape, emphasizing the flexibility of the organic molecule and the diversity of its packing by comparing with the free ligand and reported compounds.^14^,^16^ No relevant hydrogen bonds or π-stacking interactions are found in the structure, with the shortest Co^II^···Co^II^ distance at 8.962 Å.

**Fig. 2:**
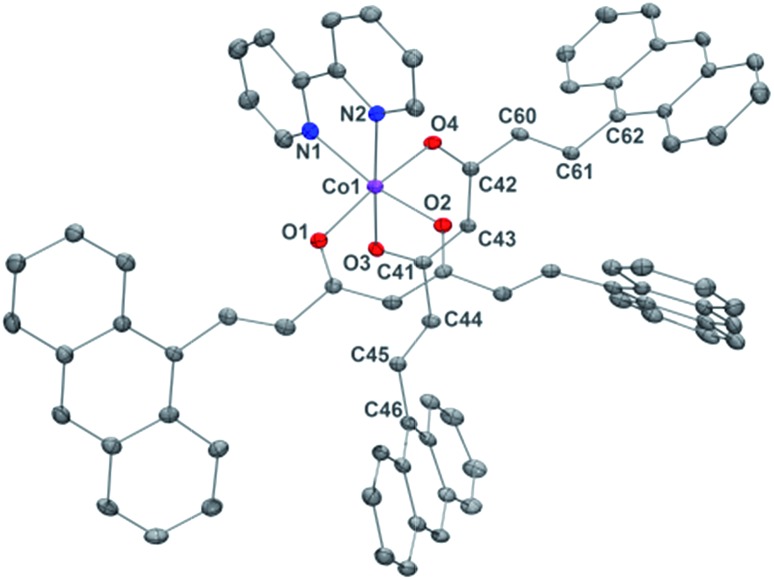
POV-Ray view of **2** with thermal ellipsoids fixed at 50%. Protons as well as the lattice chloroform molecule are omitted for clarity. Color legend: Co in magenta, O in red, N in blue and C in grey.

Compound **2**, [Co(9Accm)(2,2′-bpy)], crystallizes in the monoclinic space group *P*2_1_/*n*. The structure shows a similar compound to **1**, with a Co^II^ center bound to two molecules of 9Accm disposed in a *cis* arrangement and one molecule of 2,2′-bipyridine, now resulting in a *C*_2v_ ideal symmetry (Fig. 2). The Co–O distances between 2.012 and 2.071 Å and Co–N between 2.109 and 2.115 Å, are related to others in the literature.^19^ On the contrary, O–Co–O, N–Co–N and O–Co–N angles differ slightly with respect to **1** (see Table S3†). Similar values as in the structure of **1** are found for the C–C distances in both 9Accm ligands, with one of them presenting its two sides in a zig-zag conformation, meanwhile the other shows zig-zag and boat-shape conformations. The shortest Co^II^···Co^II^ separation is 10.105 Å and no significant supramolecular interactions can be identified, except a short C–H···O contact of the lattice chloroform molecule with O3 at 2.269 Å.

Both crystal structures could only be achieved using an X-ray synchrotron source. The flexibility of the chain observed in **1** and **2** by the different arrangements and the absence of further supramolecular interactions among neighbouring molecules could be associated with the small size of the crystals and the difficulties, observed also in related coordination compounds, of growing them.

### Studies in solution

#### Paramagnetic proton NMR

**Fig. 3:**
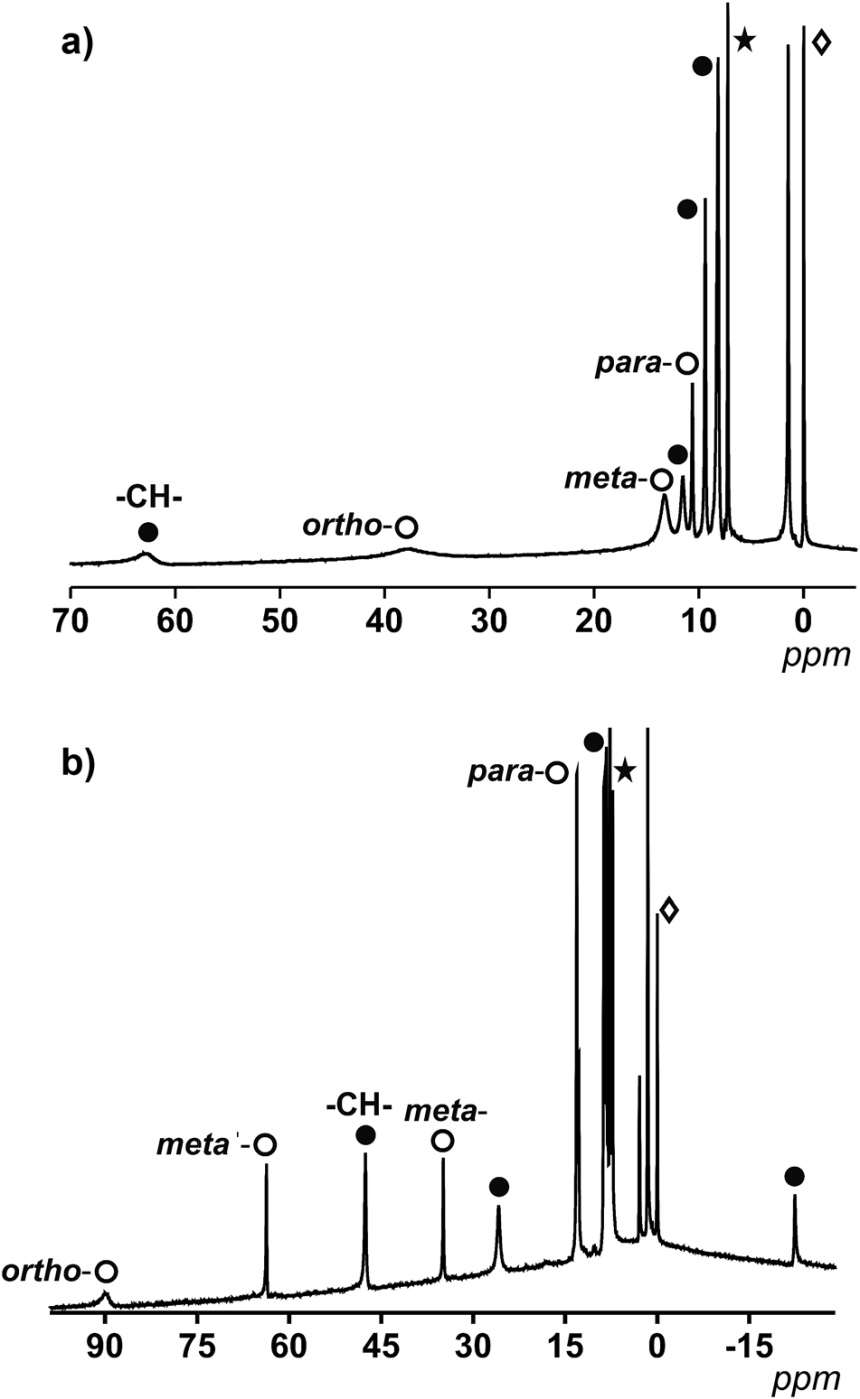
(a) ^1^H NMR spectrum of **1** in CDCl_3_ between 5–70 ppm. (b) ^1^H NMR spectrum of **2** in CDCl_3_ between 30–100 ppm. The white spheres relate to protons from py (a) or 2,2′-bpy (b) and the black spheres relate to coordinated 9Accm. *CDCl_3_ and ^[lozenge or total mark]^TMS.

^1^H NMR spectra of **1** and **2** were measured in CDCl_3_ and are shown in Fig. 3. Contrary to most paramagnetic nuclei, octahedral (Oh) Co^II^ centers display slow nuclear relaxation.^20^ Therefore, the spectral features of “[Co(9Accm)_2_]” systems are found to be sharp enough to use NMR as a diagnostic tool for their analyses in solution.^20^ To gain further insight into the paramagnetic features of **1** and **2**, their stability in solution and the effect of the geometry, additional *cis* and *trans* compounds (**3–6**) were synthesized and characterized using IR, EA and electrospray ionization. Hence, two additional *trans* compounds with formulae [Co(9Accm)_2_(3,5-(CH_3_)_2_-py)_2_] (**3**) and [Co(9Accm)_2_(dmf)_2_] (**4**), together with two *cis* compounds, [Co(9Accm)_2_(4,4′-(CH_3_)_2_-2,2′-bpy)] (**5**), and [Co(9Accm)_2_(5,5′-(CH_3_)_2_-2,2′-bpy)] (**6**) were studied in solution to gather information about the nature of most of the peaks. In addition, the available literature on mononuclear Co^II^ systems containing pyridinic and acac groups was of great relevance for the assignment of the peaks.^21^

For **1**, which displays an ideal *D*_4h_ symmetry, the number of peaks in the proton NMR reduces to eight (taking into account the overlap of some of the signals, free rotation of the anthracene groups in solution and the fast conformations that the 9Accm chain can experience), as if there was only one magnetically unique 9Accm and pyridine ligand as well. As Fig. 3a shows, compound **1** presents two distinct regions in a window of approximately 75 ppm: (i) two broad resonances in the downfield area (38 and 63 ppm, respectively) and (ii) six sharper shifts that vary in intensity between 15 and 0 ppm (upfield). The position and shape of the downfield signals relate to the closest protons to the Co^II^ center, which are the methine –CH– from the 9Accm groups and the ones in the *ortho*- position from the two pyridine molecules.^22^ The assignments of these two signals were based on previous literature^21^ and the comparison between **1** and compounds **3** and **4** (Fig. S1 and S2,† respectively).

From the data collected, the peak at 63 ppm was assigned to the –CH– of 9Accm appearing in all three compounds; meanwhile, the absence of the broad peak at 38 ppm in **4** proved a pyridinic origin. The latter, together with two other sharper peaks at 12.1 and 8.0 ppm, were related to the *ortho*-, *para*- and *meta*-protons of the pyridine molecule, respectively. The general appearance and order of the proton shifts for the coordinated py molecules suggest contact shifts *via* π delocalization as the major contributor.^23^ The rest of the signals of the upfield sector (∼10 to 0 ppm) were associated with the chain and anthracene groups from the 9Accm ligands, further away from the metallic nuclei and therefore less affected.^21^–^24^ The individual assignment of the latter could not be made however, the spectrum is consistent with the retention of the idealized symmetry of **1** in solution. The complete list of peaks for **1**, **3** and **4** is shown in Table S2.†

Fig. 3b shows the spectrum of **2**. The ideal symmetry of this system (*C*_2v_) would make the two halves of the 2,2′-bpy molecule and the two 9Accm ligands equivalent. The experiment shows that the spectrum comes close to that expected, displaying one type of 9Accm and four signals for the 2,2′-bpy (*ortho*-, *meta*-, *meta*′- and *para*-protons). Earlier publications on the subject^21^,^24^ together with the comparison of **2** and compounds **5** and **6** has allowed the assignment of the peaks. Now, the system presents a richer downfield area exhibiting sharp resonances at 89.1, 63.0, 47.0, 34.6 and 25.7 ppm with an upfield region that goes from 13.0 to –22.3 ppm. Table S3† shows the list of resonances for **2**, **5** and **6** and Fig. S3 and S4† show the spectra of **5** and **6**, respectively.

Previous literature shows a usual *ortho*-, *meta*′-, *meta*- and *para*-order (from downfield to upfield) for the proton resonances in Co^II^-(2,2′-bpy) systems. Also, former compounds showed shifts comparable to those found for compound **2**.^21^,^22^ This, together with the study of **5** and **6** allows the assessment of the two downfield shifts, at ∼90 and 63 ppm, that correspond to the *ortho*- and *meta*′-protons from the 2,2′-bpy. The following resonance at 47 ppm relates to the methine –CH– proton of the 9Accm, drastically shifted compared to that of compound **1** (which appears at 63 ppm). The following *meta*- and *para*-shifts from the 2,2′-bpy were assigned at ∼35 and ∼13 ppm, respectively, suggesting the rest of the signals (∼26, ∼13, 8.7–7.7 and –22 ppm) are of CCMoid nature (chain and anthracene groups of coordinated 9Accm) as it is indicated in Fig. 3b.

Overall, the NMR studies of **1** and **2** provide information about (i) the preservation of the molecular structures in solution, (ii) the flexibility of the chain in 9Accm and the fast free rotations of the anthracene groups, and (iii) the great influence of the paramagnetic center on the ligands upon coordination, clearly shown by the shift between the methine peaks (–CH–) of **1** and **2** (16 ppm of difference) and the display of resonances of curcuminoid nature at the highest fields present in **2** (–22 ppm). In addition, thanks to the information gathered, paramagnetic ^1^H NMR can be used to predict the *cis* or *trans* nature of future “[Co(CCMoid)_2_]” systems by the evaluation of the shift of the –CH– from the coordinated CCMoid.

#### UV-vis absorption spectra and fluorescence

The electronic spectra of **1** and **2** in distilled THF showed absorptions around 255 and 425 nm band regions (Fig. S5†). Intense bands were observed at the highest energies, related to π–π* transitions.^9^,16c Smaller broad bands, with maxima at 426 and 424 nm for **1** and **2**, respectively, were associated with the CCMoid character (π–π*) of both systems, with small hypsochromic shifts for both, **1** and **2**, compared to the free ligand, 9Accm (427 nm), due to the coordination to the metal centers.^14^,^16^ A shoulder between 300–400 nm is sometimes appreciable with maxima features characteristic to anthracene groups. In CH_2_Cl_2_, the lowest energy bands appeared now at 437 (**1**) and 428 (**2**) nm, indicating higher solvatochromic effects for **1** than **2** (Fig. S6†).16*c*

**Fig. 4:**
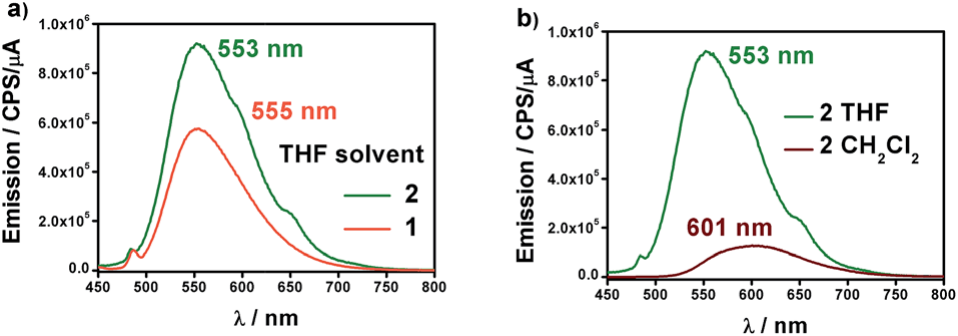
(a) Emission spectra of **1** (orange) and **2** (green) in distilled THF. (b) Emission spectra of **2** in distilled CH_2_Cl_2_ (brown) and THF (green).

Fig. 4a shows the fluorescence emission spectra of **1** and **2** in distilled THF when excited at 426 and 424 nm, respectively. The fluorescence band values were found to be 555 and 553 nm, in that order. The observed shifts are very close to the free 9Accm (*λ*_em,max_ = 555 nm), displaying similar behaviours. The shape and large Stokes shift of the bands show the CCMoid origin of the fluorescence as well as suggest small changes in the molecules following excitation, most likely due to a loss of symmetry or aggregation status.^9^,16a,c

The quantum yields of the two compounds are in sharp contrast to those of the ligand 9Accm, which exhibited stronger fluorescence emission (Fig. S7†). This fact is common in paramagnetic metal centers that normally act as quenchers displaying chelation enhancement of quenching (CHEQ) effects.^9^,^25^ Despite that, both [Co(9Accm)_2_] systems depict reasonable emissions most likely due to the number of anthracene groups per molecule, their free rotation in solution and the relatively long distance between such groups and Co^II^. The emission intensity of **1** is slightly smaller than that of **2**, a fact that is reflected in their quantum yield values, *φ*, being 0.0010 and 0.0014 for compounds **1** and **2**, respectively (Table 1); both, approximately, one order of magnitude smaller than the free 9Accm (0.010).^14^

The solvatochromic properties were explored by recording their emission spectra in CH_2_Cl_2_ and comparing with those published for free 9Accm. Fig. 4b shows as an example, the results for compound **2**. The first observation is that the emission intensities are significantly higher in THF than in CH_2_Cl_2_, indicating the additional quenching effect of the latter. In addition, there is a bathochromic effect (red shift) of ∼50 nm from THF (555 (**1**) and 553 (**2**) nm) to CH_2_Cl_2_ with the maxima now appearing at 603 and 601 nm for both compounds (Fig. S8† and 4b), respectively, and significantly shifted (∼25 nm) from the free ligand under the same conditions (577 nm).16*c* The results show solvatochromic emissions for **1** (Fig. S8†) and **2** (Fig. 4b) in a similar way to others published in the past as well as the effect of solvent polarity on the final emissions.16b,c Overall, fluorescence is qualitatively affected in the same manner for both compounds.

### Solid state properties

#### Static magnetic properties

**Fig. 5:**
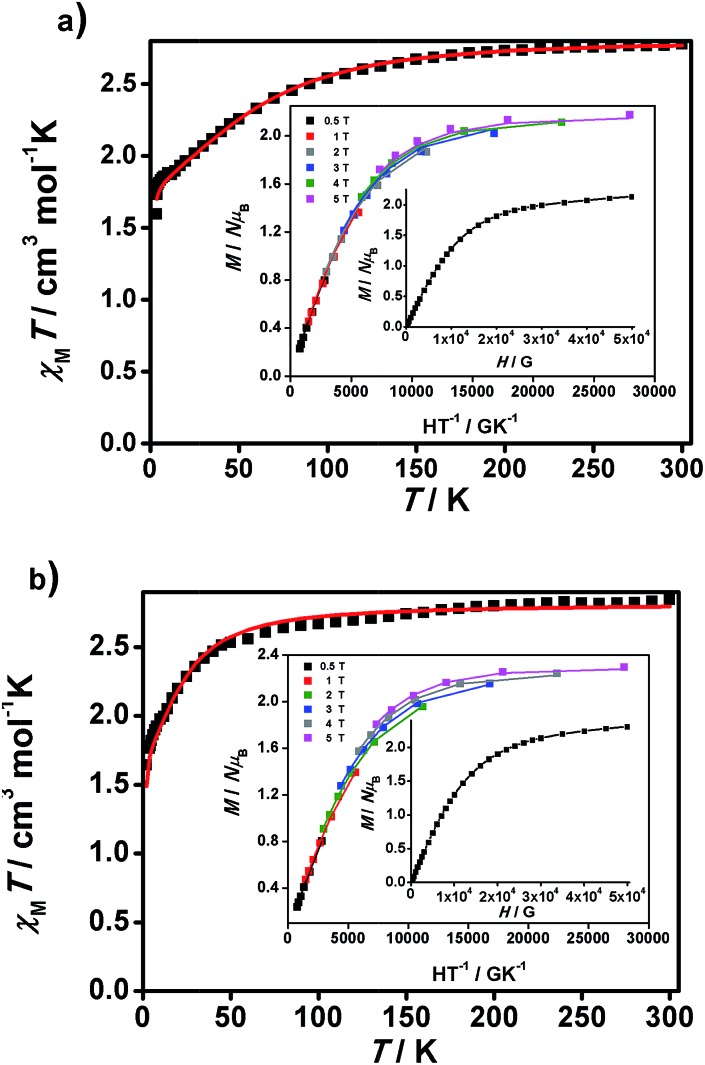
*χ*_M_ *T vs. T* graphs and insets, *M*/*Nμ*_B_*vs. H*/*T* and *M*/*Nμ*_B_*vs. H* data, for **1** (a) and **2** (b). Experimental data are shown as dots and the resulting fitting is shown by a line.

Lately a fast growing family of mononuclear Co^II^ SMMs have been described^26^ and some of us have incorporated straightforward rules to identify them.^27^ Compounds **1** and **2** follow the requirements of possible SMM candidates and therefore magnetic susceptibility was measured for polycrystalline samples of **1** and **2** using dc and ac techniques. Herein, the dc magnetic studies are presented as *χ*_M_*T vs. T*, *M*/*Nμ*_B_*vs. H* and *M*/*Nμ*_B_*vs. H*/*T* plots (Fig. 5), *χ*_M_ being the molar paramagnetic susceptibility and *N* and *μ*_B_ having the usual meaning. The temperature dependences of the *χ*_M_*T* product of **1** and **2** are displayed in Fig. 5a and b, together with their *M*/*Nμ*_B_*vs. H* plots (insets). At 300 K, the *χ*_M_*T* products of **1** and **2** are equal to 2.77 and 2.87 cm^3^ K mol^–1^, respectively, both higher than that calculated for an isolated *S* = 3/2 system (*χ*_M_*T* = 1.875 cm^3^ K mol^–1^, *g* = 2.0) due to expected spin-orbital contributions. Lowering the temperature, the *χ*_M_*T* products smoothly decrease down to 100 K and 50 K, respectively, before dropping in a smooth way for **1** to reach 1.59 cm^3^ K mol^–1^ at 3 K and more abruptly for **2**, reaching the value of 1.64 cm^3^ K mol^–1^, at 2 K. In addition, the *M vs. H*/*T* data were collected in the magnetic field and temperature ranges of 0.5–5 T and 1.8–6.8 K to determine the zero-field splitting and rhombic parameters (*D* and *E*) for both compounds. The resulting data for **1** and **2** are plotted in Fig. 5 (inset) as reduced magnetization *M*/*Nμ*_B_*vs. H*/*T*. The data were fit by diagonalization of the spin Hamiltonian matrix, using the program PHI,^28^ which allows the correlation of the experimental magnetic data of orbitally degenerate systems using multiple sources; in this case, the *χ*_M_*T vs. T* data together with the *M vs. H*/*T* results were used simultaneously. The obtained fit gave *g* = 2.26, *D* = 74.1 cm^–1^ and *E* = 1.21 cm^–1^ for **1** and *g* = 2.39, *D* = 24.1 cm^–1^ and *E* = –1.89 cm^–1^ for **2** (Fig. 5a and b).

Large *D* values were already expected from the analysis of the *M*/*Nμ*_B_*vs. H* data at 2 K, which presents saturation at the highest magnetic fields (population of the lowest *m*_s_ state) for **1** and **2**, with values close to 2 *μ*_B_ (2.13 and 2.28 *μ*_B_, respectively), lower than those expected for *S* = 3/2 (*M*/*Nμ*_B_ = 3.0 *μ*_B_, *g* = 2), indicating that there are considerable orbital contributions in both cases. Indeed, the *D* value of **1** is comparable to the highest *D* value of 80 cm^–1^ described until now by Cano *et al.*26*i*

Further analyses of the second-order anisotropy parameters (value and sign of *D* and *E*) were pursued based on eqn (1)–(3)^29^ as both anisotropic parameters are derived from the principal elements of the *D* tensor1
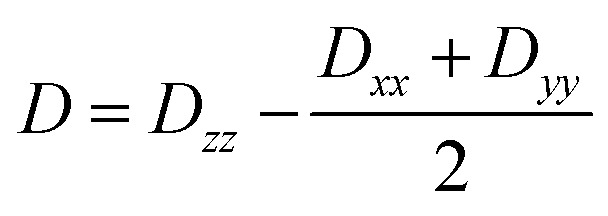

2
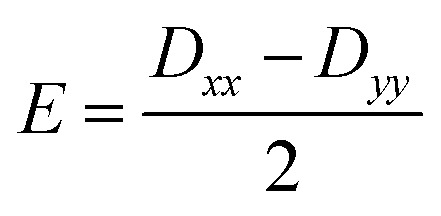
that can be estimated as follows:3
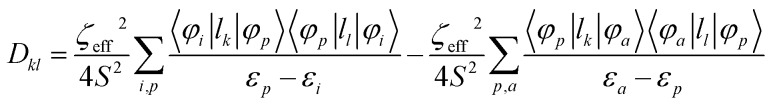
where *ζ*_eff_ is the monoatomic spin–orbit coupling constant; *l*_*k*_/*l*_*l*_ are the *x*, *y*, *z* components of the angular momentum operator, and *ε* indicates the molecular orbital energy with the sub-index *i*, *p* or *a*, that indicate double-occupied, singly-occupied or empty orbitals, respectively. Intuitively, following eqn (3), small excitation energies (*δ* in Table 1) also result in orbital energy differences, giving rise to large diagonalized *D*_*ii*_ values (*δ* ≈ *ε*_*p*_ – *ε*_*i*_ and *D*_*ii*_ = *D*_*xx*_, *D*_*yy*_ or *D*_*zz*_).^27^ In the case of a single Co^II^ ion (d^7^) in a pseudo-octahedral coordination with d2*xz* d2*yz* d1*xy* d1*z*2 d1*x*2–*y*2 orbital occupation (like **1** and **2**), the first excitation energies, *δ*, correspond to transitions between the beta d_*xz*_ or d_*yz*_ orbitals and the beta d_*xy*_ orbital, which are small (see Table 1), explaining the high values of *D* for both compounds (where **1** is one of the highest found in the literature). On the other hand, taking into account the above excitation energies, **1** and **2** are described as easy-plane systems instead of being easy-axis due the symmetry of the orbitals involved (change in absolute *m*_L_ value in the first excitation) that makes operator matrix *D*_*xx*_ and *D*_*yy*_ terms be predominant.^30^ Thus, the (*D*_*xx*_ + *D*_*yy*_)/2 term in eqn (1) will be larger than the *D*_*zz*_ term, resulting in positive *D* values (all terms of eqn (3) are strictly negative). These qualitative arguments have been confirmed by CASSCF/NEVPT2 calculations including spin orbit effects (Table 1) agreeing with the positive signs and large values of *D* found in the fittings of **1** and **2**, respectively.

**Table 1:** *φ* stands for quantum yield. Values of *D* and *E* (all in cm^–1^) for the *S* = 3/2 ground state of compounds **1** and **2** calculated with CASSCF and NEVPT2 (values in parentheses) methods (see Computational details section). The last two columns give the first excitation energy *δ* and *Δ* (in cm^–1^) without and after including spin–orbit effects, respectively. The *Δ* value corresponds to the energy difference between the ground and excited Kramers′ doublets

	*φ*	*D*_fitting_	*E*_fitting_	*g*_fitting_	*D*_calc_	*E*_calc_	*δ*_calc_	*Δ*_calc_
**1**	0.0010	74.1	1.21	2.26	167.1 (146.5)	24.0 (25.6)	405 (463)	227 (214)
**2**	0.0014	24.1	–1.89	2.39	71.6 (50.2)	7.8 (6.8)	775 (1095)	152 (113)

#### Dynamic magnetic properties

The ac magnetic susceptibility of **1** and **2** below 5 K was investigated in the presence of external dc fields, as no out-of-phase signals were observed in zero-field. Experiments at a variable frequency of up to 1480 Hz were first performed at different magnetic fields (0.05, 0.1, 0.15, 0.2 and 0.5 T for **1** and 0.03, 0.05, 0.07 and 0.1 T, in the case of **2**) to determine the most convenient dc field for the study of the magnetization dynamics of each compound. Fig. S9 and S10† show the resulting *χ*′′_M_*vs.* frequency plots, in which a maximum is observed at all fields in the case of **1**, while only at the higher fields and close to the maximum frequency in the case of **2**. The optimal fields were defined as 0.15 and 0.07 T for **1** and **2**, respectively.

**Fig. 6:**
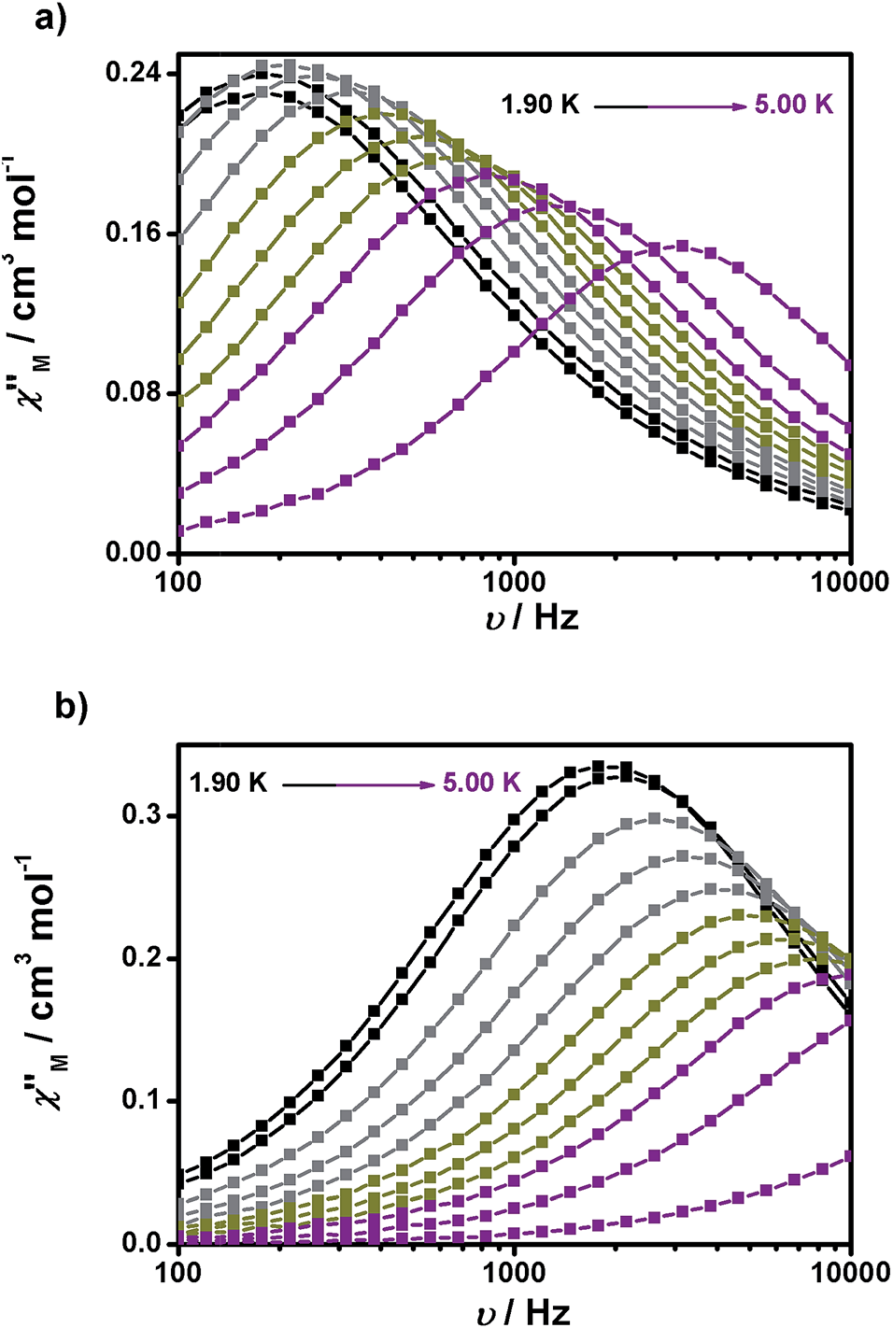
Frequency dependence of the out-of-phase (*χ*′′_M_*vs. ν*) susceptibility for **1** (a) and **2** (b) under 1500 and 700 Oe dc fields, respectively.

Experiments at a variable frequency were then repeated in the extended 100 Hz to 10 kHz range at these dc fields and at temperatures in the range of 1.9 to 6 K. The characteristic frequency dependence of the in-phase (χ′_M_) and out-of-phase (χ′′_M_) susceptibilities for SMM behaviour is clearly observed in both cases (Fig. 6a and b, respectively, as well as Fig. S11 and S12†).

**Fig. 7:**
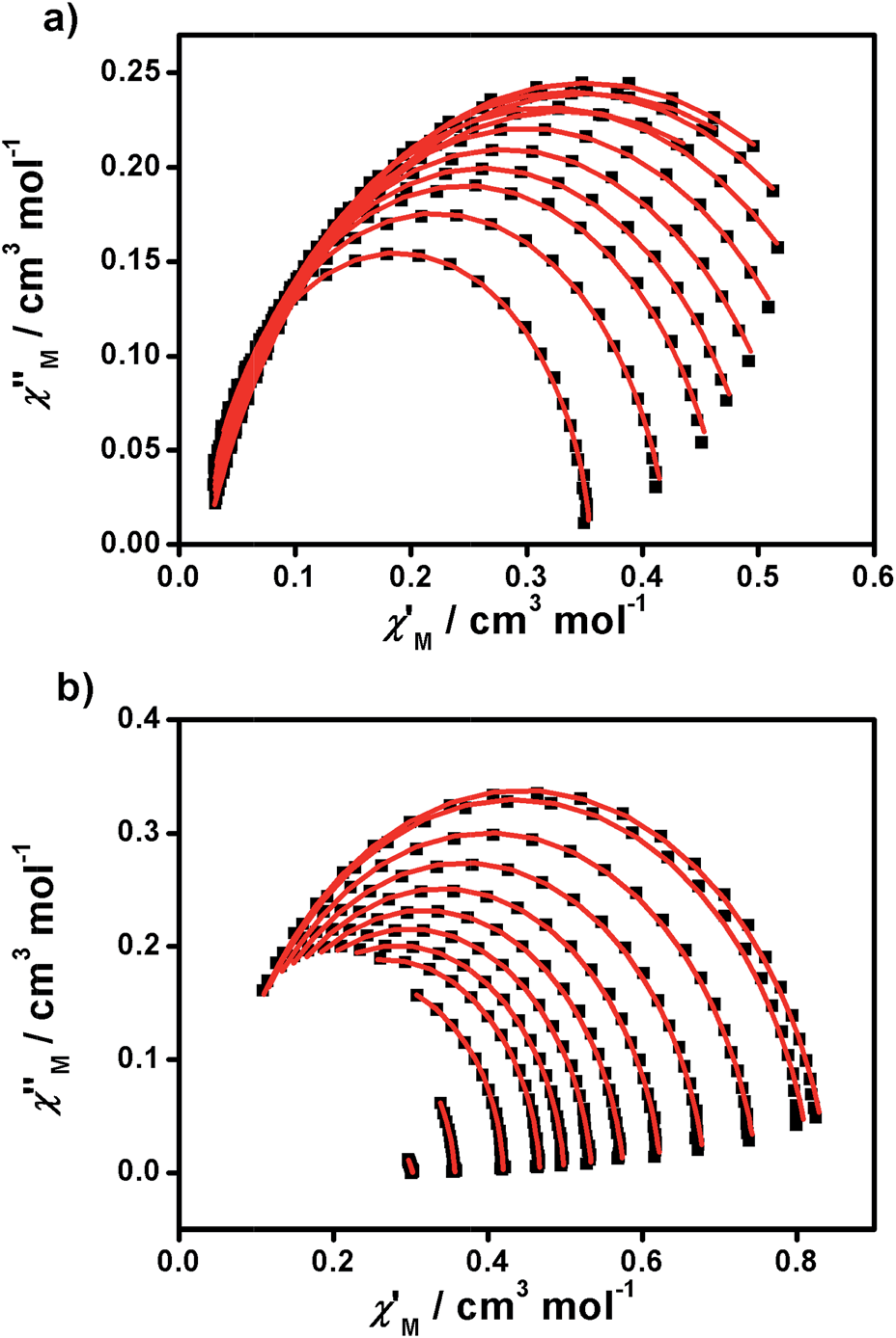
Cole–Cole plots of **1** (a) and **2** (b) measured from 1.9 to 5.0 K under 1500 and 700 Oe dc fields, respectively.

From the above experiments, Cole–Cole diagrams were extracted at the same temperature range (Fig. 7a and b), exhibiting typical semi-circular shapes. These data were fitted to the Cole–Cole expressions using the C-Cfit program,^31^ affording values of the characteristic relaxation time *τ* in the range 0.01–0.20 s^–1^ for **1** and 0.01–0.30 s^–1^ for **2**, supporting the existence of a single relaxation process in each case.

From here, the spin-lattice relaxation rate *τ*^–1^ was determined at each given temperature. The complete modelling of it, that is to say dependence *τ*^–1^*vs. T*, can be performed following eqn (4).^32^4
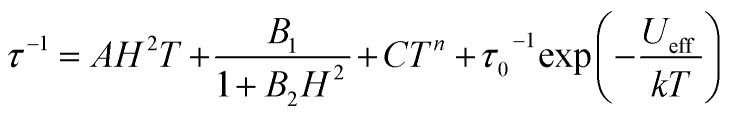



The terms in eqn (4) refer to direct relaxation, quantum tunnelling, Raman and Orbach relaxation mechanisms, in that order. Quantum tunnelling contributions are not relevant26*c* and Orbach processes are not considered because the *ab initio* calculations indicate that the first excited states are much higher in energy than the measured barrier. Hence, Raman and direct relaxation mechanisms were the only two expressions used in the simulation (eqn (5)).

5 *τ*^–1^ ≈ *A*′*T* + *CT*^*n*^

**Fig. 8:**
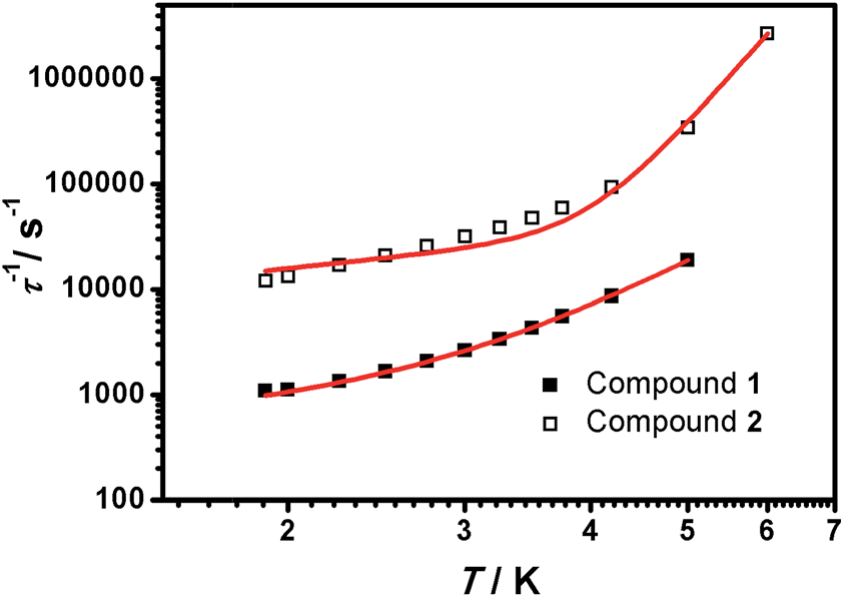
*τ* ^–1^ *vs. T* plots of **1** (■) and **2** (□) measured from 1.9 to 5.0 K under 1500 and 700 Oe dc fields, respectively.

The simulated data using eqn (5) is shown in Fig. 8. The extracted *A*′, *C* and *n* values are depicted in Table 1. To provide further analysis and with the aim of introducing a library of novel magnetic parameters, comparison of these data with published ones for other 3d mononuclear Co^II^ SMMs (Table S4†) shows that the *A*′ value for **1** (447 s^–1^ k^–1^ at 0.15 T) is comparable to previously derived values^32^,^33^ although the value for **2** (6688 s^–1^ k^–1^ at 0.15 T) is one order of magnitude higher than the available data until now. Nonetheless, the comparison and interpretation of *A*′ are not trivial, depending on several parameters,^32^ where the scarce information available restricts further conclusions. The case of the Raman term is similar, where we conclude that the *C* values found for **1** and **2** are similar to published Fe^II^ systems^32^ and again, the highest in contrast with the other Co^II^ SMMs studied this way in the literature.26*c* However, here the effect of solid dilution may play a relevant role and therefore numbers should be evaluated with caution. Our *n* factors, on the other hand, are of the order of others, being 5 for **1** and 7.5 for **2**,^32^,^33^ and reinforcing the idea that direct and Raman mechanisms are operative. Here again, the appreciable magnetic differences between the two compounds, **1** and **2**, should be highlighted, even though they share a similar ligand environment.

#### Theoretical results

Calculated second-order anisotropy parameters and excitation energies for compounds **1** and **2** are collected in Table 1. The calculated *D* and *E* values are in qualitative agreement with the fitted values showing large positive *D* values for both compounds, three times larger in the case of **1**. It is usual to expect larger calculated values in comparison with the fitted experimental data, because such spin relaxation mechanisms depending on the lattice effects are not considered in single-molecule calculations. These facts (sign and dimension of *D*) relate to the Jahn–Teller effect that causes distortions, breaking the orbital degeneracy (assuming a perfect Oh coordination), where, as explained above, small energy differences between the ground and first excited state (*δ*) affect *D* in a great manner, reflected as small denominator values in eqn (3). The small values of such energy gaps contribute to the uncertainty determining *D* (thus, the energy gap, *δ*, is so small that the systems are close to a degenerate ground state) making the first-order spin–orbit contributions also relevant.^27^,^34^ Also, previously noticed CASSCF-type calculations generally overestimate *D* values, perhaps also caused by the mentioned limitations of the spin Hamiltonian for a near-degenerate system and the lack of inclusion of some spin polarization mechanism (tunneling and collective effects) in the calculations.^27^

The origin of the large anisotropy in first-row mononuclear transition metal complexes is the presence of low-lying spin–orbit free excited states (CASSCF/NEVPT2 energies without spin–orbit contributions, *δ* in Table 1) with close energies to the ground state. Thus, systems showing a distorted geometry (in this case, pseudo-octahedral) due to the Jahn–Teller effect with respect to an ideal degenerate d^7^ octahedral configuration are perfect candidates to have close low-lying excited states.

As mentioned above using a simple single-determinant wavefunction as a model, the first excitation energies should correspond to transitions from the beta d_*xz*_ or d_*yz*_ orbitals to the beta d_*xy*_ orbital that would degenerate in the octahedral symmetry (t_2g_). This fact results in large contributions to the *D* value (see *δ*, Table 1) although they must be corrected by including the first-order spin–orbit contributions (see *Δ*, Table 1). Indeed, by doing so, it is clear why compound **2** displays the largest excitation energy *δ* but the smallest *D* value compared to **1** (*Δ* value is smaller for **2**, see Table 1). Therefore, a reliable orbital explanation for the differences in the *D* values of **1** and **2** must include the relative energies of the non-degenerate orbitals (d_*xz*_, d_*yz*_ and d_*xy*_) taking into account geometrical distortions and the presence of two different ligands (py/9Accm (**1**) and 2,2′-bpy/9Accm (**2**)). Nevertheless, basic qualitative explanations are not trivial, because the orbital energies are controlled by the subtle interplay of many parameters (different metal–ligand distances and ligand–metal–ligand angles for the two types of ligands). Thus, our DFT studies (see details in the Photoemission section) show that for **1**, a small splitting of the three orbitals was obtained with the d_*xy*_ orbital displaced to the intermediate position among the t_2g_ orbitals, meanwhile in **2**, a larger splitting was found with the d_*xy*_ orbital positioned at the highest energy. Altogether, such variations agree with the values from the fitting and explain the difference between **1** and **2**.

#### AFM deposition studies

AFM experiments were performed with deposits of **1** and **2** on highly oriented pyrolytic graphite (HOPG) and silicon (Si(100)) wafers. The experiments were performed with a double aim: the study of their affinity with the above mentioned surfaces and information on their stability from later photoemission experiments. Spin-coating experiments using CH_2_Cl_2_ solutions of **1** and **2** were performed using both substrates, HOPG and Si(100). Blanks using exclusively the solvent at the same conditions were performed for each experiment (Fig. S13†).

Depositions on freshly cleaved HOPG were performed at 500 rpm for 30 seconds; three drops of the solution were added to the surface at regular intervals (∼10 s). The HOPG experiments display the affinity of **1** and **2** for such a surface due to the π–π interactions of the anthracene groups with the substrate at room temperature.^35^ At 10^–4^ M, AFM images show the formation of multiple aggregates of molecules with heights between 1.0–1.2 nm for **1** and 1.2–1.6 nm for **2** (Fig. S14 and S15†), with average heights corresponding to piles of 1–2 molecules for **1** and **2** on the HOPG surfaces (values estimated from the crystallographic data).

Due to the closeness of the aggregates, further experiments were performed to clarify the formation of the layer(s) underneath such assemblies. After obtaining an AFM image in tapping mode, the operation was changed to contact mode for both molecules. As we described in the past,16*b* the AFM tip swept the molecules from the substrate due to the higher vertical force applied in contact mode. Afterward, the topographic mode was back to tapping mode and a larger scale was chosen in order to image the area where molecules were removed (Fig. S16†). The difference between the vertical size on the side of the hole and the undisturbed layer, on the other side, provides valuable information on the formation of the layer(s) and heights. Such experiments were successfully carried out for **2** as is shown in Fig. S16.† The collected data was similar to that described before, indicating the absence of multilayers on the surface of the HOPG substrate. All the attempts to gather the same information with compound **1** failed and final images were too vague to provide clear pictures of the surfaces. Toward photoemission experiments, full coverage of the surface was accomplished by increasing the number of solution drops of **1** and **2** on the HOPG surfaces.

Similar experiments using Si(100) presented clear aggregation even at higher concentrations, being impossible to accomplish full coverage of the surface and therefore further photoemission experiments. Such behaviour directly relates with solvent evaporation effects (CH_2_Cl_2_), the conjugated nature of the two compounds and probably the deposition methodology, emphasizing once again the higher affinity of the compounds toward the HOPG.

#### Photoemission

**Fig. 9:**
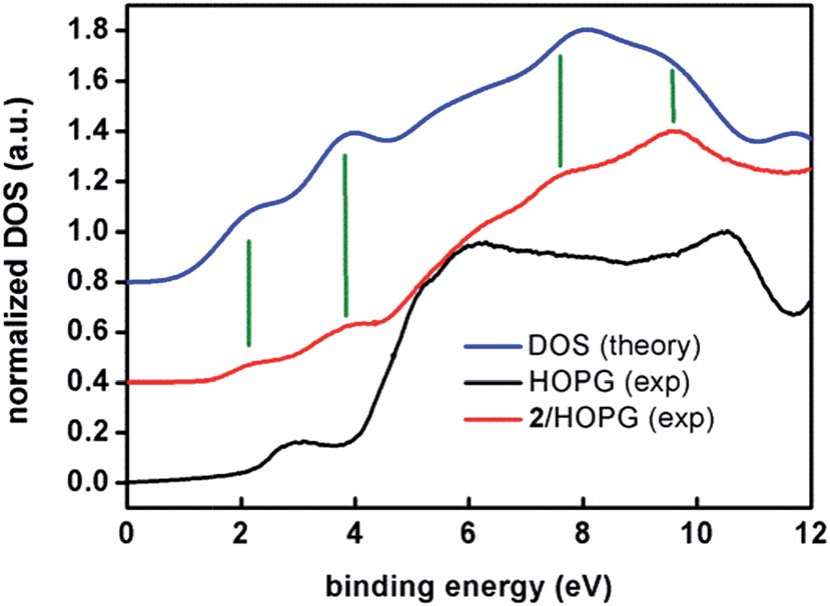
Experimental UPS (ultra-violet photo-emission spectroscopy) density of states spectra of a spin-coated film of **2** on HOPG (red) and of a freshly cleaved HOPG surface (black) compared to the DFT calculated spectrum (blue). The high-resolution UPS spectra were acquired with a pass energy of 5 eV in UHV and at room temperature. The binding energies are referred to the Fermi level of the system (*E*_F_ = 0 eV). The spectra have been normalized to their maxima and shifted in the vertical scale for clarity. The DFT calculated DOS spectrum has been shifted by 3.1 eV in order to level the HOMO. Vertical green lines have been included to guide the eye comparing experimental and calculated bands.

XPS experiments on a film of **2** spin-coated on HOPG allowed the identification of the spin–orbit splitting lines and shape of the corresponding satellites comparable to the electronic configuration of Co^II^ (Fig. S17†). Further analyses of the sample also allowed the identification of C (sp^2^), O and N as expected from the crystal structure and bulk analyses. Fig. 9 shows the density of states (DOS) spectrum measured by means of UPS on spin-coated films of **2** on HOPG (red) compared to the calculated DOS spectrum (blue, Gaussian code^36^ with the B3LYP functional^37^ and the TZV basis^38^). The DOS of the clean substrate, a freshly cleaved HOPG surface (black), is also shown. The energy reference (0 eV) is set to the Fermi level of the experimental system, which has been previously determined with an *in situ* cleaned Au(111) crystal.^39^

Note the remarkable agreement between the experimental and calculated features at 2.1 and 3.8 eV that correspond to two sets of eight and seventeen molecular orbitals, respectively. These two first sets of orbitals contain mostly π anthracene orbitals. The e_g_ and t_2g_ orbitals are in the higher binding energy of the first and second band, respectively. Features at 7.6 and 8.6 eV are also reproduced by the calculations. The broad feature at about 6 eV is observed in both experimental and calculated spectra but it lies within the large feature arising from the HOPG substrate. Therefore, as a conclusion, both experiments, XPS and UPS, show the expected patterns for compound **2**, confirming the stability of the sample under such conditions.

XPS experiments for compound **1** on the other hand, showed a clear absence of N on the HOPG surface and ambiguous results from the Co^II^ analysis. This is probably due to the loss of py molecules during the deposition procedure, proving that compound **2** is a more robust system upon spin-coating, and clarifying the AFM experimental results for both systems. Importantly, this points out the necessity of photoemission studies on nanostructured systems toward their correct analysis.

## Conclusions

In summary, this work reports the first two crystallographically characterized mononuclear Co^II^-CCMoid coordination compounds in the literature. Both systems, **1** ([Co(9Accm)_2_(py)_2_]) and **2** ([Co(9Accm)_2_(2,2′-bpy)]), exhibit octahedral environments, containing two CCMoid ligands (9Accm) that bind one Co^II^ center together with two pyridine molecules or one 2,2′-bpy group, giving as a result *trans* (**1**) and *cis* (**2**) dispositions of the 9Accm ligands in the final arrangements. The use of microwave assisted reactions provided high yields and pure compounds. The “quasi-isomers” display comparable features and allow the study of the structural/magnetic/fluorescence similarities but also they show differences in solution and in the solid state. Paramagnetic ^1^H NMR studies of **1** and **2** show the stability of the systems in solution and allow the recognition of *cis*/*trans* Co-CCMoids by the downfield shift of the methine proton (–CH–) of the coordinated 9Accm ligands. Furthermore, moderate emissions in the visible region (related to the anthracene groups of 9Accm) have been found for both species in organic solvents despite the partial fluorescence quenching that both systems present given by the paramagnetic nature of the metal. **1** and **2** show solvatochromic effects with similar fluorescence yields. In the solid state, the two systems exhibit single-molecule magnet behaviour, albeit only under applied dc fields, and constitute the newest additions to the limited family of mononuclear Co^II^ hexacoordinated SMMs. Compound **1** presents one of the highest positive *D* values (*D* = +74 cm^–1^) found for mononuclear Co^II^ systems and compound **2** shows only about a third of this value (*D* = +24 cm^–1^). This fact emphasizes the magnetic repercussion that slight variations of the coordination sphere around the Co^II^ center have. These studies have been corroborated by CASSCF/NEVPT2 calculations, from which the positive *D* values for both systems have been obtained, the anisotropy being larger for **1** due to the existence of low-lying excited states closer in energy to the ground state. Finally, the deposition of **1** and **2** on HOPG and Si(100) substrates has been characterized. AFM images show the formation of aggregates of **1** and **2** on HOPG, showing the affinity of both species for such a substrate, although XPS and UV photoemission studies demonstrate that only compound **2** is robust enough to form stable thin films on HOPG. For such a system, the UV photoemission results are in excellent agreement with the theoretical calculations.

Altogether, **1** and **2** present major differences in their magnetic performance in solution and in the solid state, meanwhile their fluorescence properties are comparable in solution. On the other hand, studies in solution depict the stability of both systems but the deposition on HOPG (by the use of spin-coating) points out the necessity of careful characterization of molecules on surfaces, **1** being unstable under the experimental conditions and **2** being the most robust system among the two described. In addition, we have introduced additional techniques such as paramagnetic ^1^H NMR, fluorescence and UV photoemission within the field of SMMs toward further analyses of functional molecular materials and therefore, their consideration in other areas related to nanoscience.

## Supplementary Material

Supplementary information

Crystal structure data
