# Supplementary material for: Multiscale study of mononuclear CoII SMMs based on curcuminoid ligands
Source: Chem Sci. 2016 Jan 7;7(4):2793–803. doi: 10.1039/c5sc03298a (PMC6054041; doi:10.1039/c5sc03298a)
Supplement: Supplementary file 1 [file SC-007-C5SC03298A-s001.pdf]

Electronic Supplementary Material (ESI) for Chemical Science.
This journal is © The Royal Society of Chemistry 2016

Supplementary Information to the paper:

Multiscale Study of Mononuclear Co^{II} SMMs based on Curcuminoid Ligands

Raúl Díaz-Torres,[a] Melita Menelaou,[a] Olivier Roubeau,[b] Alessandro Sorrenti,[c] Guillem Brandariz-de-Pedro,[a] E. Carolina Sañudo,[a] Simon J. Teat,[d] Jordi Fraxedas,[ef] Eliseo Ruiz[ag] and Núria Aliaga-Alcalde[h*]

[a] *Departament de Química Inorgànica, Universitat de Barcelona, Diagonal 645, 08028 Barcelona, Spain.*
[b] *Instituto de Ciencia de Materiales de Aragón (ICMA), CSIC and Universidad de Zaragoza, Plaza San Francisco s/n 50009 Zaragoza, Spain.*
[c] *CSIC-ICMAB (Institut de Ciència dels Materials de Barcelona) Campus de la Universitat Autònoma de Barcelona, 08193 Bellaterra, Spain.*
[d] *Advanced Light Source, Lawrence Berkeley National Laboratory, Berkeley, California 94720, USA.*
[e] *CN2 - Institut Catala de Nanociencia i Nanotecnologia, Campus UAB, 08193, Bellaterra (Barcelona), Spain.*
[f] *CSIC - Consejo Superior de Investigaciones Cientificas, ICN2 Building, 08193 Bellaterra (Barcelona), Spain.*
[g] *Institut de Química Teòrica i Computacional, Universitat de Barcelona, Diagonal, 645, 08028 Barcelona, Spain.*
[h] *ICREA (Institució Catalana de Recerca i Estudis Avançats) & CSIC-ICMAB (Institut de Ciència dels Materials de Barcelona) Campus de la Universitat Autònoma de Barcelona, 08193 Bellaterra, Spain.*

List of contents:

Materials and Methods. All experiments were carried out in aerobic conditions using commercial grade solvents for the synthesis of the two compounds. Solvents were distilled for the UV-vis, fluorescence and nano-structuration studies. 9Accm was synthesized according to the procedure described elsewhere.[8]

Crystallography. Due to the crystal size and shape, data of sufficient quality could only be obtained using synchrotron radiation sources. Data were obtained at 100 K on a yellow plate of dimensions 0.12 × 0.05 × 0.01 mm^3 and an orange plate of dimensions 0.07 × 0.04 × 0.02 mm^3 respectively for compounds 1 and 2, in both cases on a Bruker APEXII-CCD diffractometer on Advanced Light Source beamline 11.3.1 at Lawrence Berkeley National Laboratory from a silicon 111 monochromator (λ = 0.7749 Å). Data reduction and absorption corrections were performed with SAINT[12] and SADABS.[13] Both structures were solved and refined on F^2 using the SHELXT suite.[14] Crystallographic and refinement parameters are provided in Table S1, while selected bond distances and angles are given in Tables S2 and S3. All details can be found in the supplementary crystallographic data for this paper in CIF format with CCDC numbers 1411573-1411574. These data can be obtained free of charge from the Cambridge Crystallographic Data Center via https://summary.ccdc.cam.ac.uk/structure-summary-form.

Physical Measurements. C, H and N analyses were performed with a Perkin-Elmer 2400 series II analyzer. Mass spectra were recorded in $CHCl_3$/MeCN (1:1) using matrix assisted laser desorption ionization with time of flight (MALDI-TOF) mass spectrometer (4800 Plus MALDI TOF/TOF (ABSciex - 2010)). DHB stands for 2,5-dihydroxybenzoic acid. Infrared spectra (4000-400 cm^{-1}) were recorded from KBr pellets on a Bruker IFS-125 FT-IR spectrophotometer. Fluorescence emission spectra were carried out on a Horiba-Jobin-Yvon SPEX Nanolog-TM. Fluorescein was used as a fluorophore standard for quantum yield experiments. AFM images were captured in tapping mode using a Multimode AFM attached to Nanoscope IV electronics

(Digital Instruments, Santa Barbara, CA). Single beam silicon oxide AFM probes were used (T300R-W series, VistaProbes, Phoenix, AZ) with a nominal spring constant of 40nN/nm. In order to preserve the structure of the sample, the free amplitude of the AFM probe was kept to a minimum and images where acquired at the maximum amplitude set-point. Scan rate was set to 0.7 Hz and resolution was 512x512 pixels. Blanks containing the solvent, CH_2Cl_2 (from same butch used for the compounds) following rigorously the exact procedure were performed for each experiment; images of those are depicted in the Supplementary Information, SI. dc and ac magnetic measurements were performed with Quantum Design MPMS-5S and MPMS-XL magnetometers either at the Universitat Barcelona or at the Physical Measurements unit of the Servicio General de Apoyo a la Investigación-SAI, Universidad de Zaragoza. ac susceptibility data in the range 100-10000 Hz were obtained at this same facility using the ac-susceptibility option of a Physical Properties Measurement System from Quantum Design.

Computational Details. CASSCF/NEVPT2 calculations were performed to extract zero-field splitting parameters of the Co^{II} cations using the whole molecular structure of the complexes with version 2.9.1 of the ORCA program[16] using the TZVP basis set proposed by Ahlrichs[17] and the auxiliary TZV/C Coulomb fitting basis sets.[18] The active space consisted of five orbitals occupied by seven electrons to describe the static correlation. Dynamic correlation effects were included through n-electron valence state perturbation theory (NEVPT2) method. For the calculations of the magnetic anisotropy, the contributions from ten quartet and forty doublet excited states were considered. Quasi-degenerate perturbation theory was used to add the spin-orbit coupling elements to the Hamiltonian matrix. Diagonalization of such matrix provides the final complex eigenstates.

Photoemission. Ultra-violet Photoemission Spectroscopy (UPS) and X-ray Photoelectron Spectroscopy (XPS) experiments were performed at a base pressure of 10^{-10} mbar and room temperature using a SPECS PHOIBOS 150 hemispherical analyzer equipped with a 2D CCD detector and using monochromatic HeI (21.22 eV) and Al$K\alpha$ (1486.6 eV) radiation as excitation sources, respectively. The samples were prepared by spin-coating a solution of **1** and **2** in CH_2Cl_2 on freshly cleaved HOPG substrates at 300 rpm and introduced in the fast-entry lock vacuum system shortly after deposition to avoid contamination. After pumping overnight samples were transferred to the analysis chamber. Beam damage has to be characterized when exposing molecular films to intense UV beams.[19,20] The stability of the films has been checked by accumulating several spectra at the same point. We do not observe changes in films of **2** after acquiring three consecutive spectra. The spectra shown here correspond to the first acquired ones on a new region of the sample. Prior to the UPS experiments, exploratory XPS was performed on the films. Films of **2** showed the presence of N1s and Co2p lines, apart from the C1s and O1s lines, with the characteristic shape of a Co^{II} electronic configuration. However, for films of **1** no N1s lines could be detected, so that such films were not considered for the UPS experiments.

Syntheses of “*cis*” and “*trans*” compounds.

Figure S1. ^{1}H NMR spectrum of **3** in $CDCl_3$.

Figure S2. ^{1}H NMR spectrum of **4** in $CDCl_3$.

Figure S3. ^{1}H NMR spectrum of **5** in $CDCl_3$.

Figure S4. ^{1}H NMR spectrum of **6** in $CDCl_3$.

^{1}H NMR comparison of “*cis*” compounds.

^{1}H NMR comparison of *“trans*” compounds.

Figure S5. Comparison of the UV-Vis spectra of complexes **1** and **2** in THF.

Figure S6. Comparison of the UV-Vis spectra of complexes **1**, **2** and **9Accm** in CH_2Cl_2.

Figure S7. Comparison of the Fluorescence emission of complexes **1**, **2** and **9Accm** in THF.

Figure S8. Solvatochromic emission for compound **1** in CH_2Cl_2 and THF.

Figure S9. Representation χ''_M vs. ν at different magnetic fields H_{DC} of compound **1**.

Figure S10. Representation χ''_M vs. ν at different magnetic fields H_{DC} of compound **2**.

Figure S11. Representation χ''_M vs. T at different frequency for compound **1**.

Figure S12. Representation χ''_M vs. T at different frequency for compound **2**.

Figure S13. AFM experiment; example of blank.

Figure S14. AFM experiments (tapping mode) of compound **1**.

Figure S15. AFM experiments (tapping mode) of compound **2**.

Figure S16. AFM experiments (contact mode – tapping mode) of compound **2**.

Figure S17. XPS experiments of a film of **2** spin-coated on HOPG.

Table S1. Crystal data and structure refinement for compounds **1** - **2**.

Table S2. ^{1}H NMR peaks of compounds **1**, **3** and **4**.

Table S3. ^{1}H NMR peaks of compounds **2**, **5** and **6**.

Table S4. Comparison of magnetic data A', C and n published and this work.

Syntheses of “*cis*” and “*trans*” compounds

trans compounds:

Compound [Co(9Accm)$_2$(3,5-(CH$_3$)$_2$-py)$_2$] (**3**) and compound [Co(9Accm)$_2$(dmf)$_2$] (**4**) were synthesized in the same way that compound (**1**) but 3,5-(CH$_3$)$_2$-py and DMF were used in the case of compounds (**3**) and (**4**), respectively.

cis compounds:

Compound [Co(4,4'-Dimethyl-2,2'-dipyridyl)(9Accm)$_2$] (**5**) and compound [Co(5,5'-Dimethyl-2,2'-dipyridyl)(9Accm)$_2$] (**6**) were synthesized in the same way that compound (**2**) but using 4,4'-Dimethyl-2,2'-dipyridyl (for **5**) and 5,5'-Dimethyl-2,2'-dipyridyl (for **6**).

[Co(9Accm)$_2$(3,5-(CH$_3$)$_2$-py)$_2$] (**3**)

Yield: 89 %. Analc. Calcd. for $C_{84}H_{64}CoN_2O_4 \cdot 0.3C_7H_9N \cdot 0.3H_2O$ (1261,13 g·mol^{-1}): C 81.91; H 5.8; N 2.55. Found: C 81.57; H 5.48; N 2.63. IR Data (KBr, cm^{-1}): 3419, 3046, 3021, 3006, 2920, 1640, 1566, 1499, 1452, 1408, 1348, 1264, 1161, 968, 881, 844, 790, 736, 703, 605, 538, 446. MALDI+ (DHB) (m/z): 1010.3 ([Co(9Accm)$_2$]$^+$) and 1032.3 ([Co(9Accm)$_2$ + Na]$^+$).

[Co(9Accm)$_2$(dmf)$_2$] (**4**)

Yield: 93 %. Analc. Calcd. for $C_{76}H_{60}CoN_2O_6 \cdot 1C_3H_7NO \cdot 0.5H_2O$ (1237,44 g·mol^{-1}): C 76.62; H 5.53; N 3.39. Found: C 76.21; H 5.38; N 2.99. IR Data (KBr, cm^{-1}): 3426, 3043, 3021, 2925, 1652, 1561, 1504, 1442, 1405, 1378, 1348, 1262, 1156, 1096, 970, 881, 844, 792, 733, 671, 602, 538, 444. MALDI+ (DHB) (m/z): 1010.3 ([Co(9Accm)$_2$]$^+$) and 1032.3 ([Co(9Accm)$_2$+ Na]$^+$).

[Co(9Accm)$_2$(4,4'-(CH$_3$)$_2$-2,2'-bpy)] (**5**)

Yield: 97 %. Analc. Calcd. for $C_{72}H_{58}CoN_2O_4 \cdot 0.4C_3H_7NO$ (1102,59 g·mol^{-1}): C 79.68; H 5.55; N 3.05. Found: C 79.48; H 5.25; N 3.34. IR Data (KBr, cm^{-1}): 3419, 3048, 3021, 2915, 1672, 1615, 1558, 1506, 1437, 1408, 1348, 1296, 1168, 1084, 1044, 968, 874, 842, 736, 622, 602, 528. MALDI+ (DHB) (m/z): 718.2 ([Co(9Accm)(4,4'-Dimethyl-2,2'-dipyridyl)]$^+$) and 1032.3 ([Co(9Accm)$_2$+ Na]$^+$).

[Co (9Accm)$_2$(5,5'-Dimethyl-2,2'-dipyridyl)] **(6)**

Yield: 86 %. Analc. Calcd. for $C_{72}H_{58}CoN_2O_4$ (1073.37 g·mol^{-1}): C 80.51; H 5.44; N 2.61. Found: C 80.60; H 5.04; N 2.55. IR Data (KBr, cm^{-1}): 3426, 3046, 3024, 2922, 1672, 1630, 1558, 1506, 1437, 1346, 1161, 985, 968, 879, 847, 782, 736, 605, 538. MALDI+ (DHB) (m/z): 718.2 ([Co(9Accm)(5,5'-Dimethyl-2,2'-dipyridyl)]$^+$) and 1032.3 ([Co(9Accm)$_2$+ Na]$^+$).

Figure S1. ^{1}H NMR spectrum of **3** in $CDCl_3$ between -10 - 70 ppm.

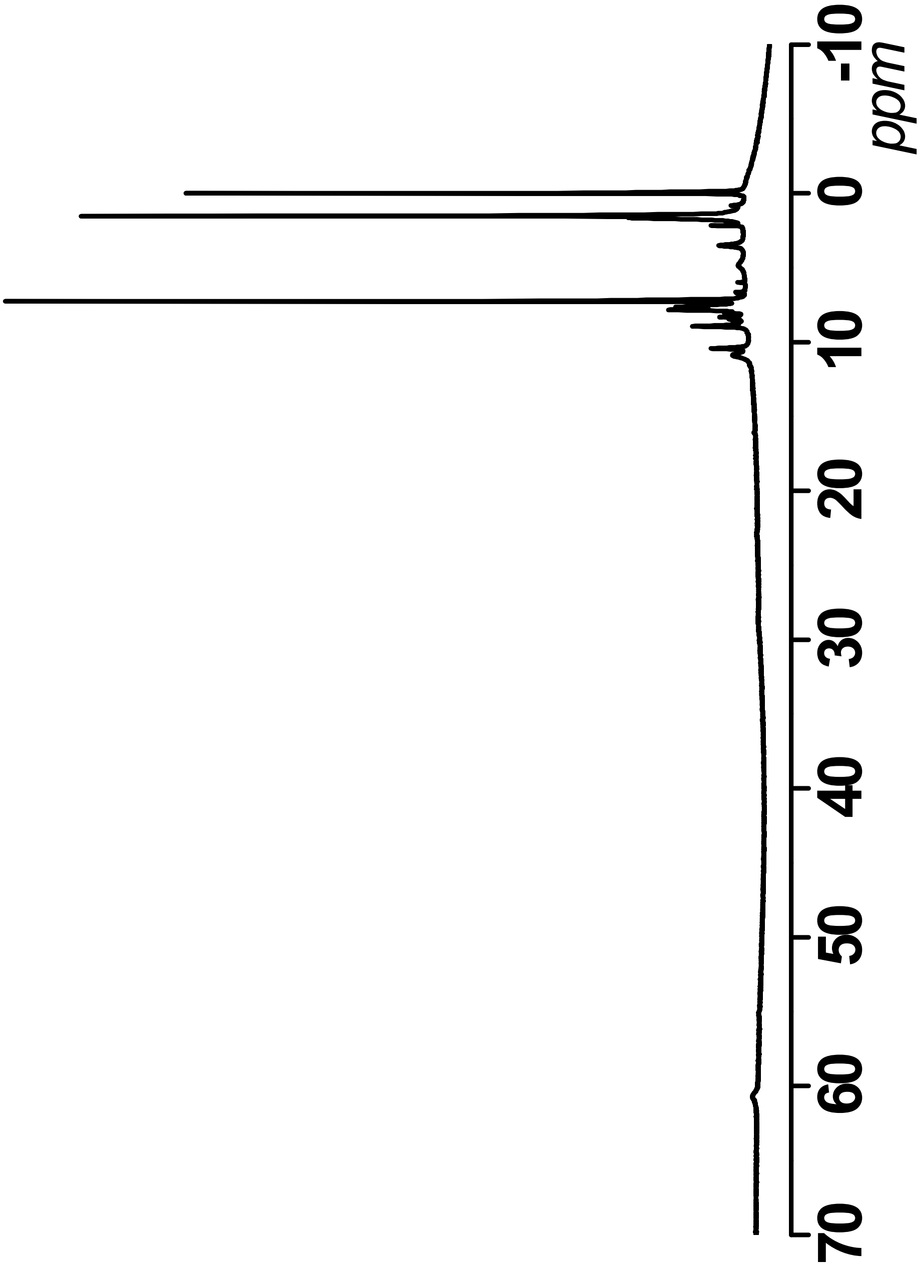

Figure S2. ^{1}H NMR spectrum of **4** in $CDCl_3$ between -10 - 70 ppm.

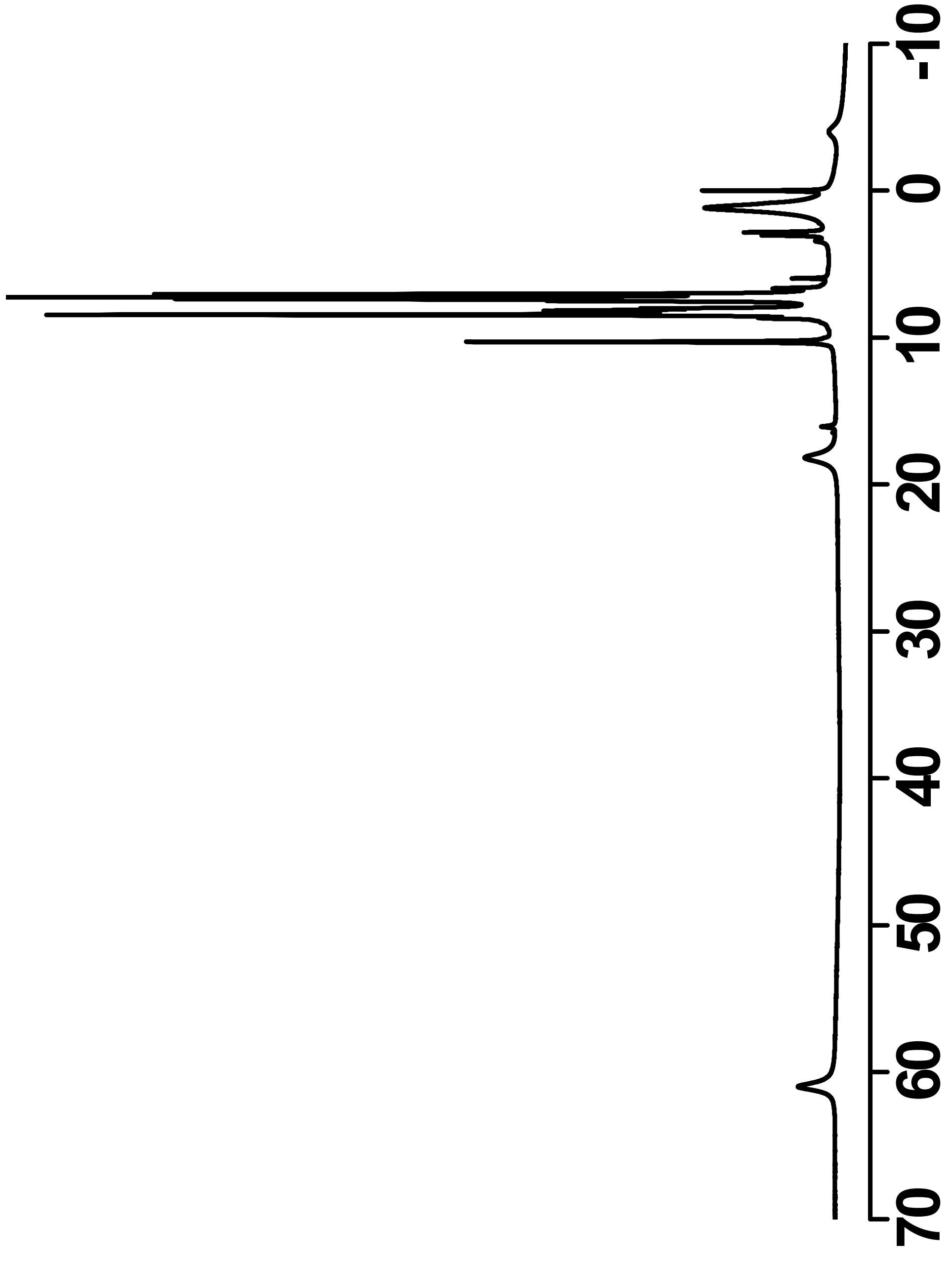

Figure S3. ^1H NMR spectrum of **5** in $CDCl_3$ between -25 - 95 ppm.

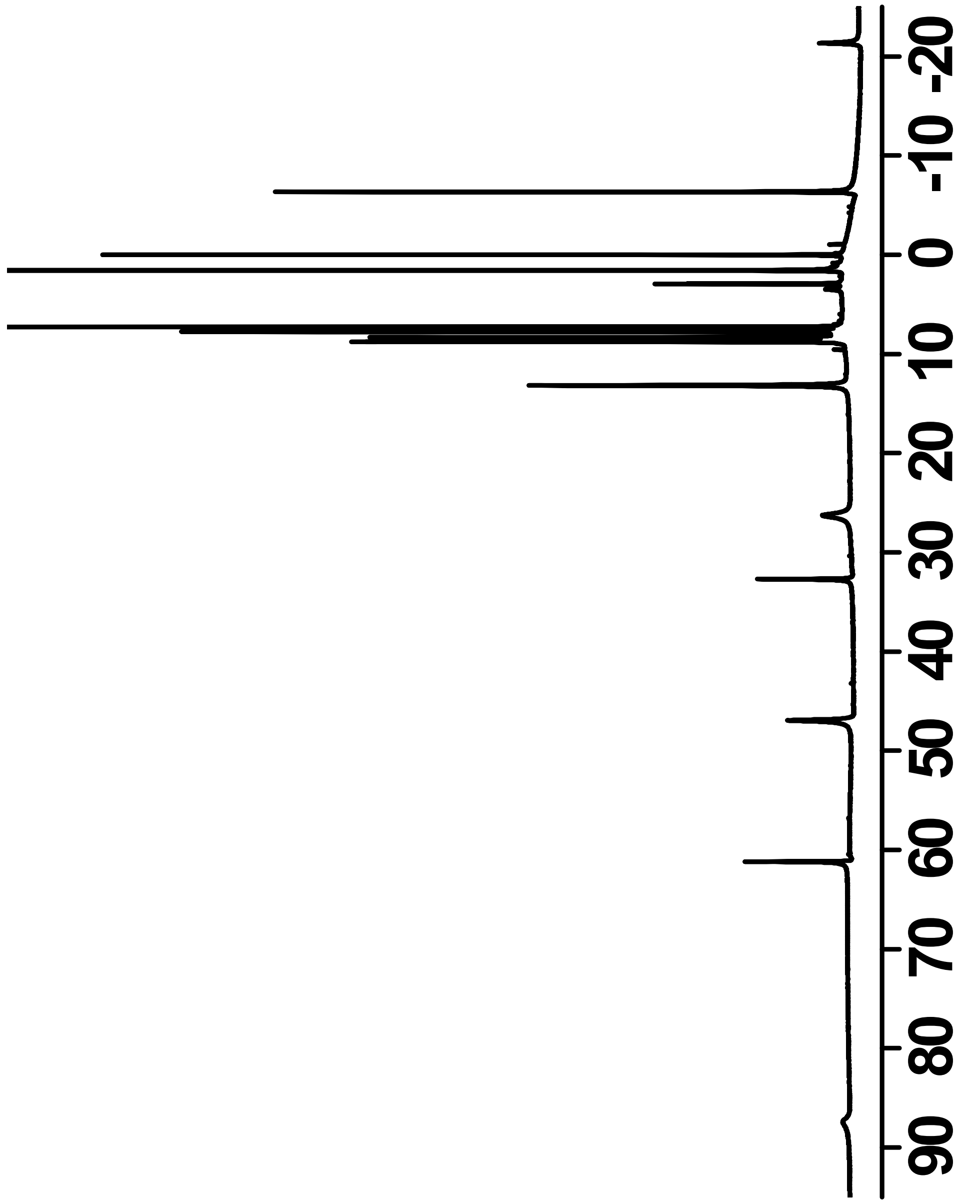

Figure S4. ^1H NMR spectrum of **6** in $CDCl_3$ between -25 - 95 ppm.

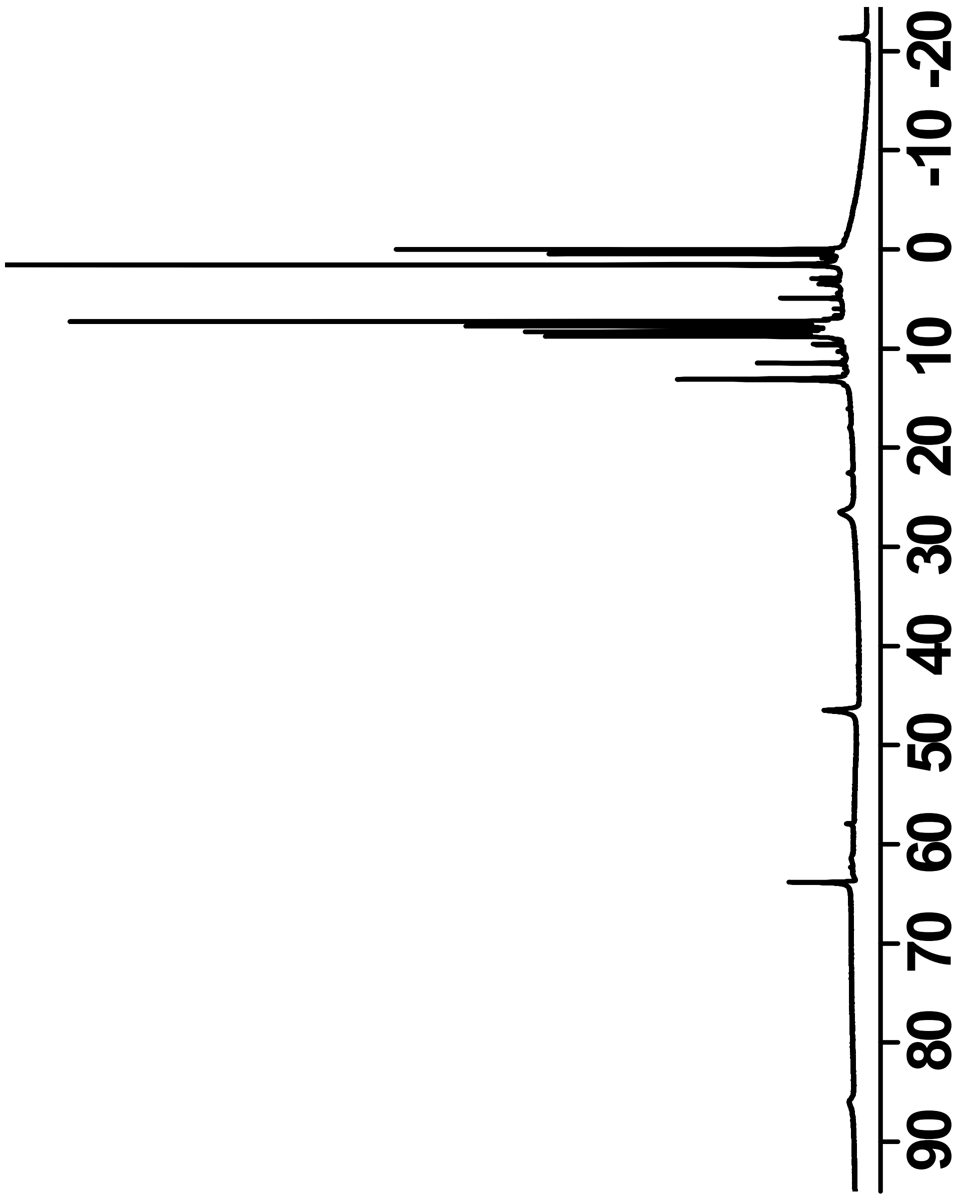

^{1}H NMR Comparison of *"cis"* compounds

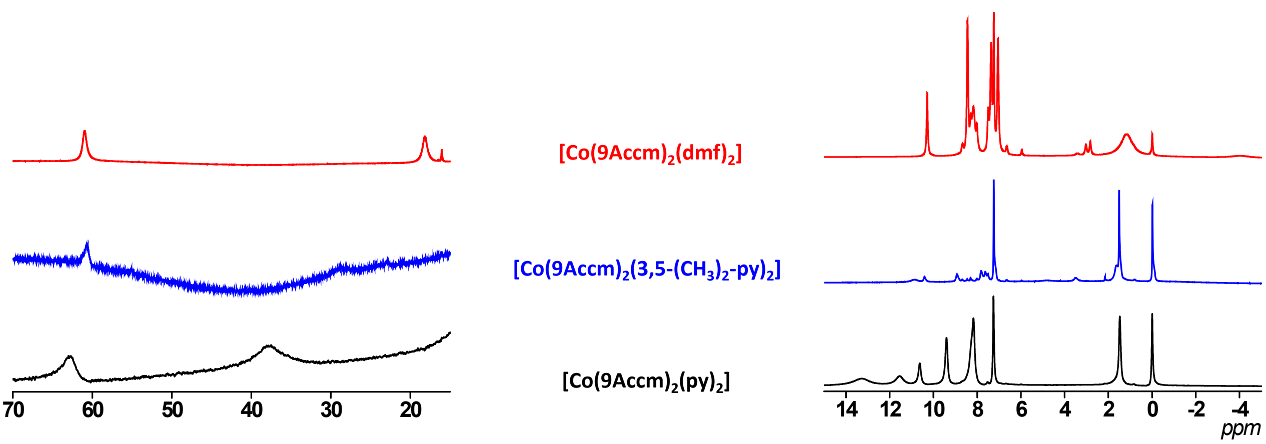

^{1}H NMR Comparison of "*trans*" compounds

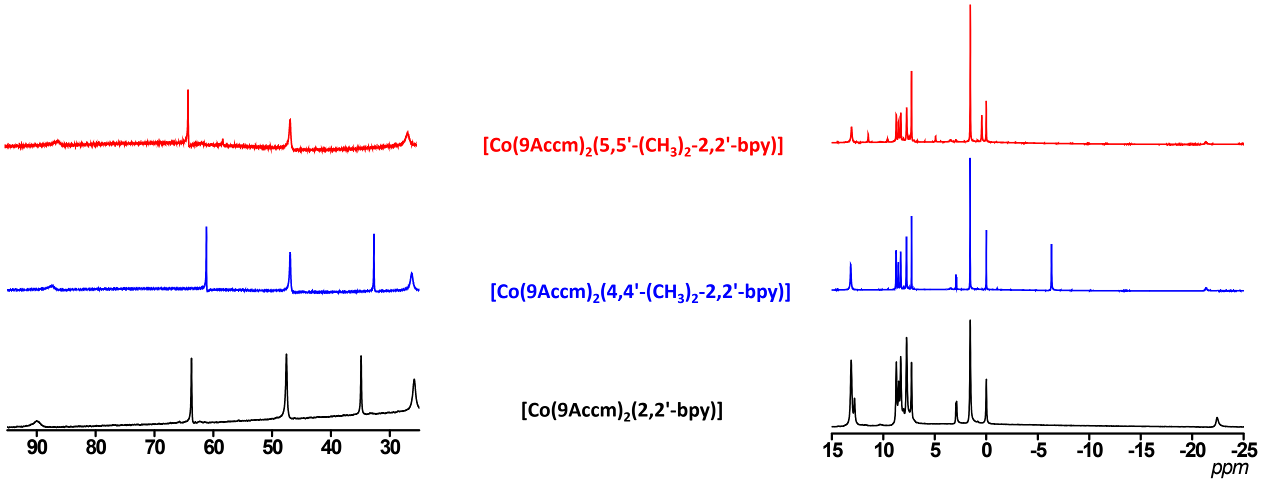

Figure S5. Comparison of the UV-Vis spectra of complexes **1** (orange) and **2** (green) in THF.

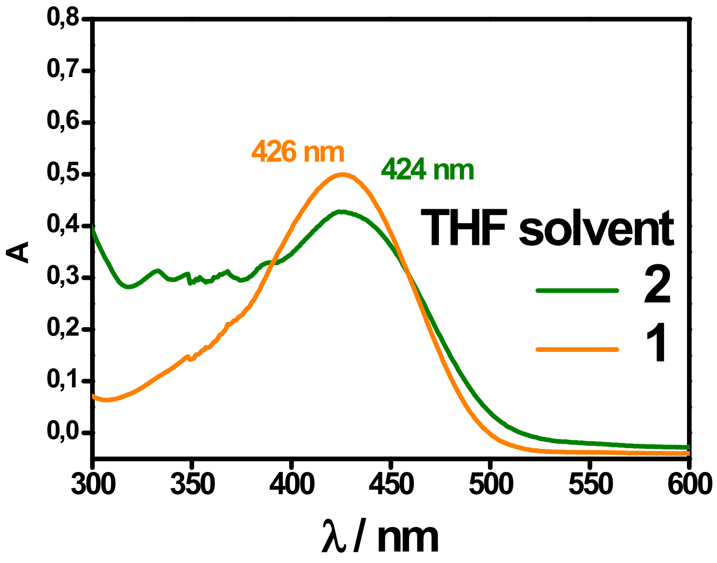

Figure S6. Comparison of the UV-Vis spectra of complexes **1** (orange), **2** (green) and **9Accm** (red) in CH_2Cl_2.

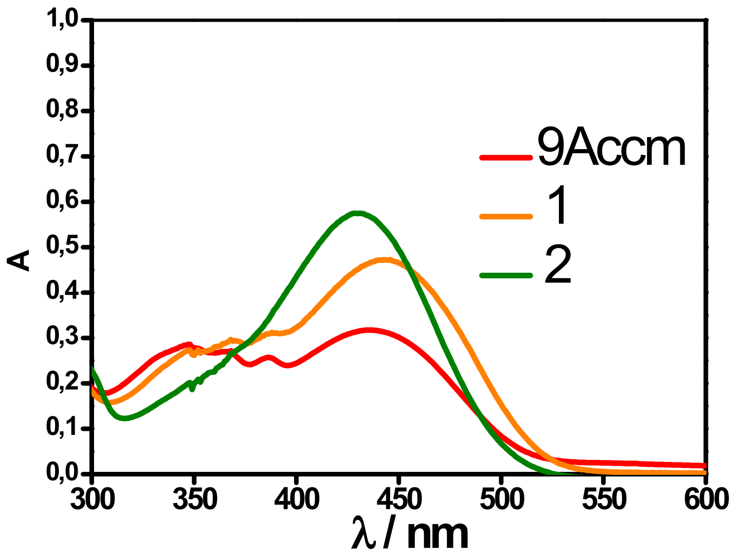

Figure S7. Comparison of the Fluorescence emission of complexes **1** (orange), **2** (green) and **9Accm** (red) in THF.

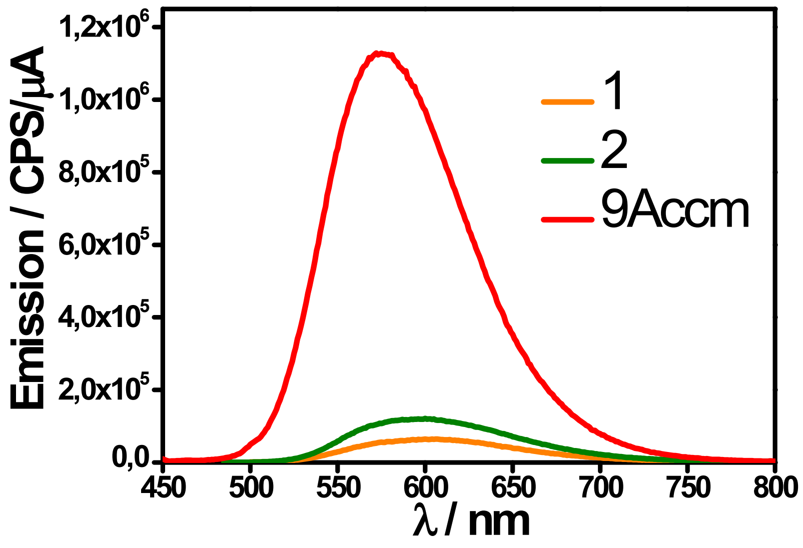

Figure S8. Solvatochromic emission for compound **1** in CH_2Cl_2 (orange) and THF (brown).

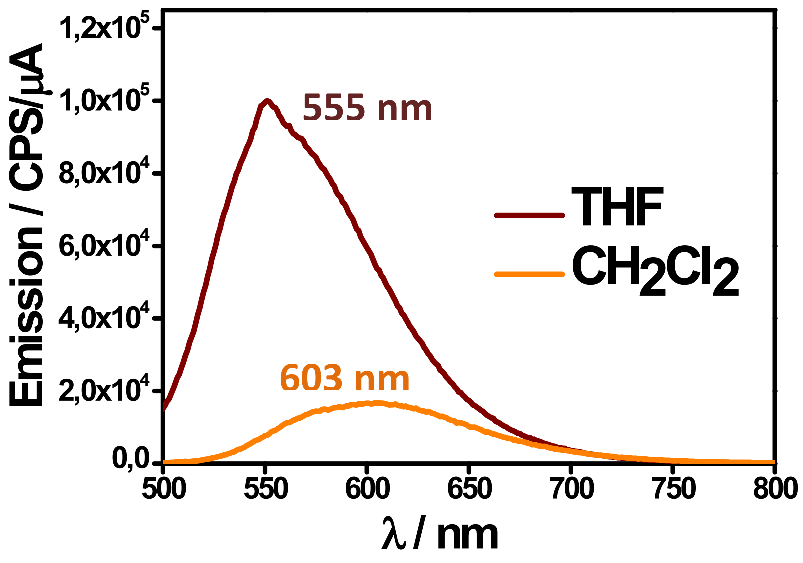

Figure S9. Representation χ''_M vs. ν at different magnetic fields H_{DC} of compound **1**.

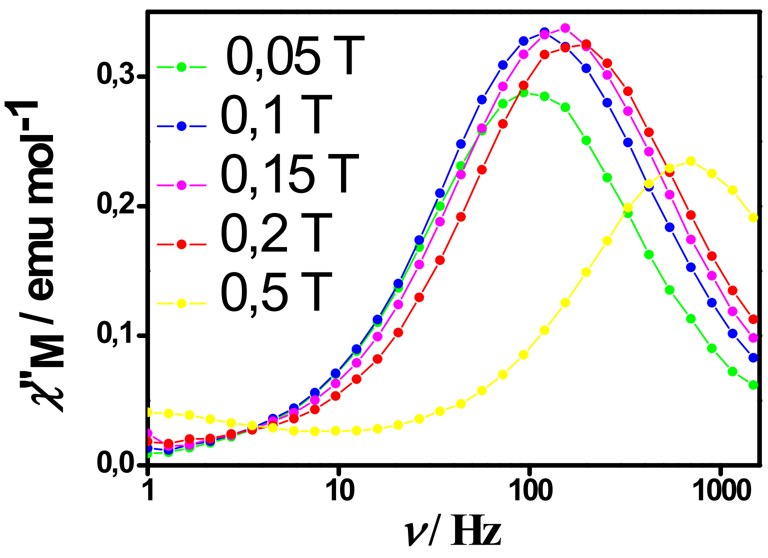

Figure S10. Representation χ''_M vs. ν at different magnetic fields H_{DC} of compound **2**.

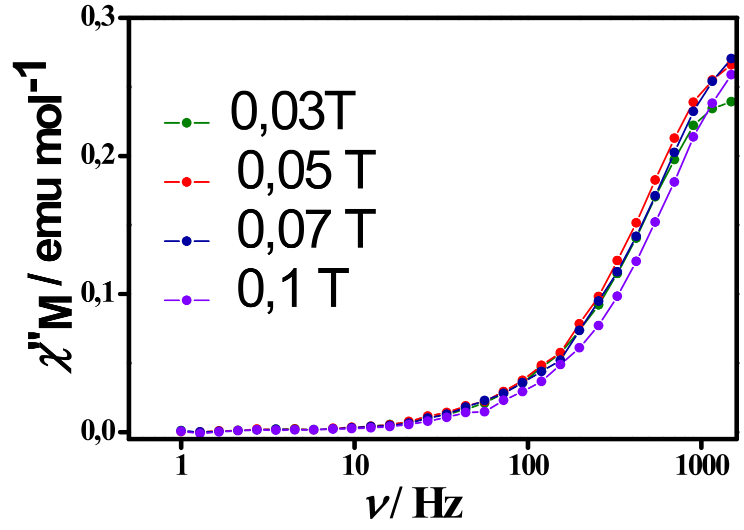

Figure S11. Representation χ"$_M$ vs. T at different frequency for compound **1**.

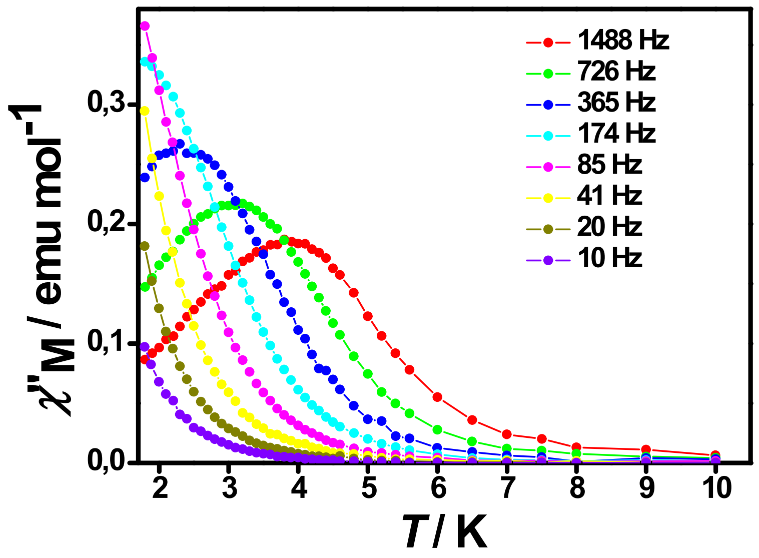

Figure S12. Representation χ"$_M$ vs. T at different frequency for compound **2**.

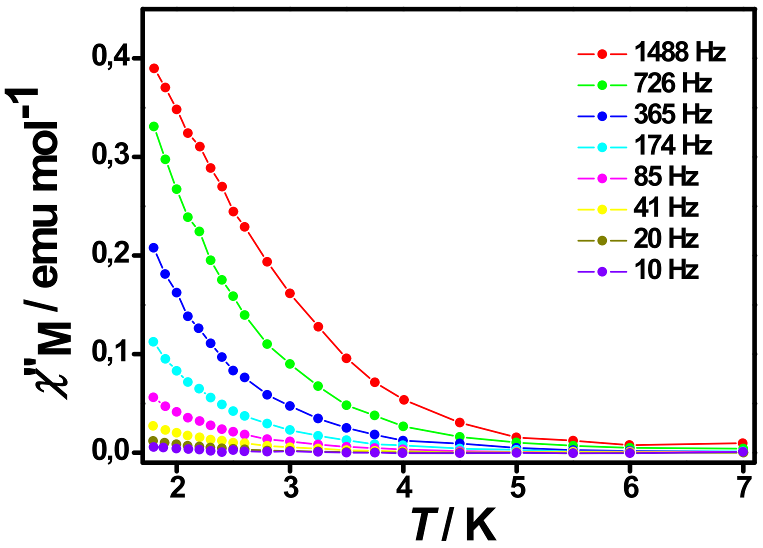

Figure S13. AFM experiment; example of a blank.

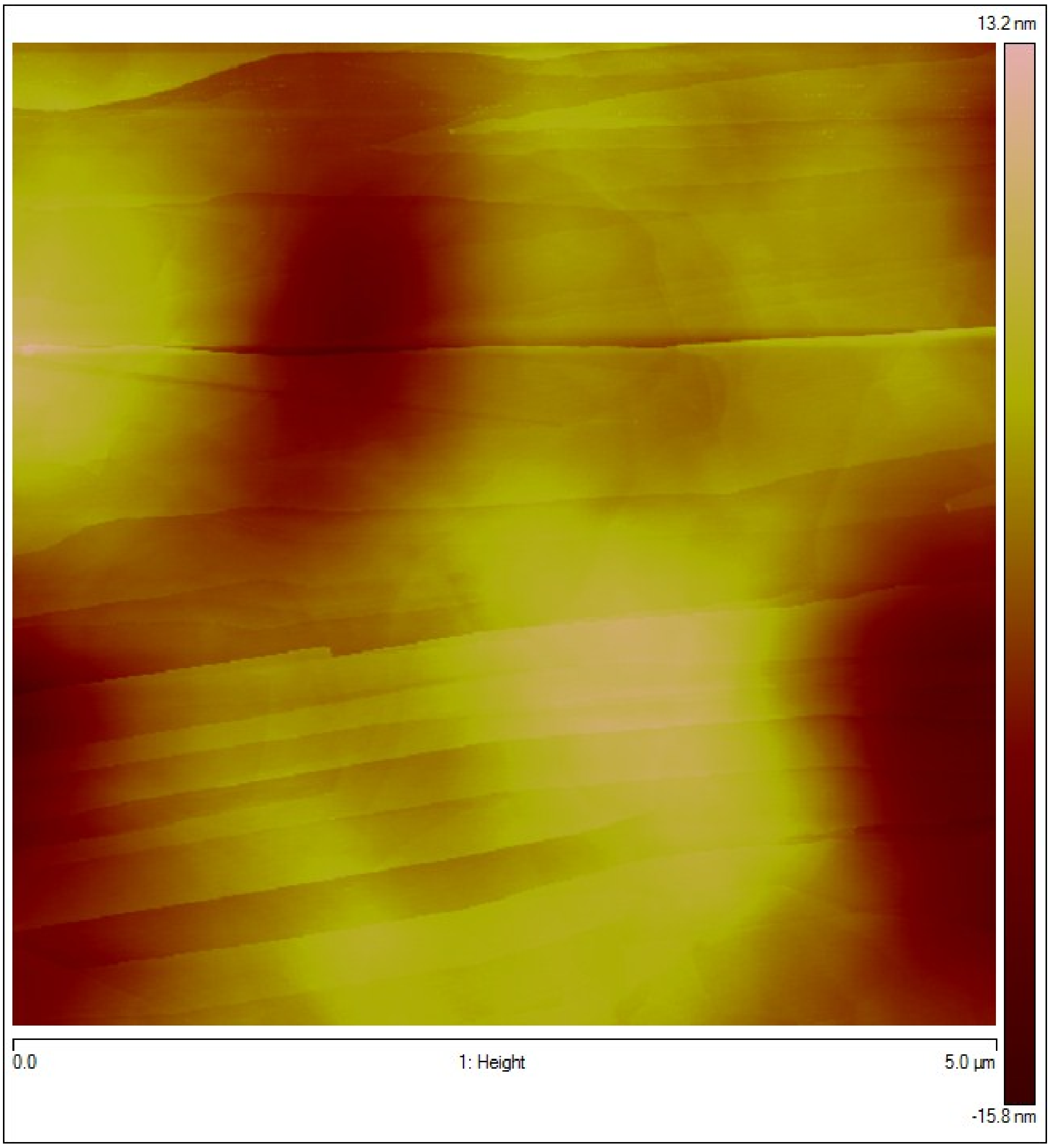

Figure S14. AFM experiments (tapping mode) of compound **1**.

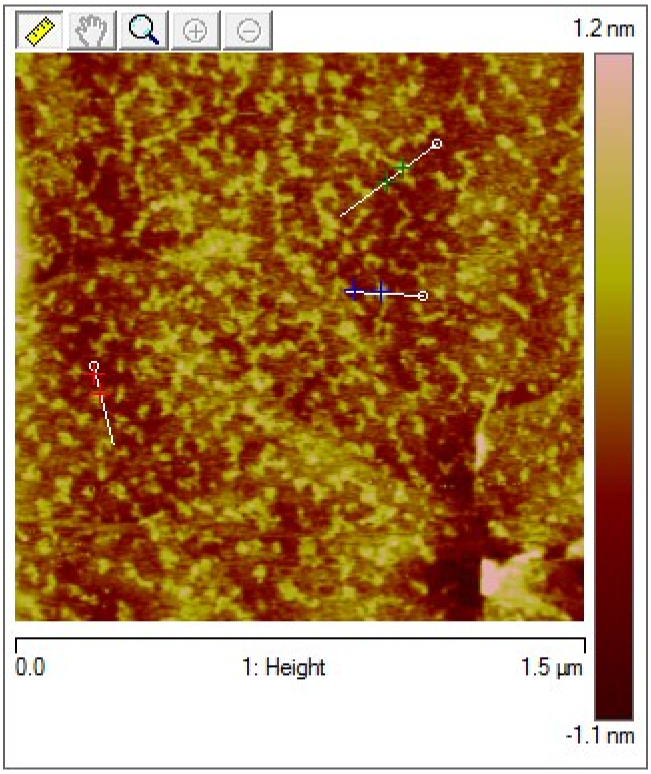

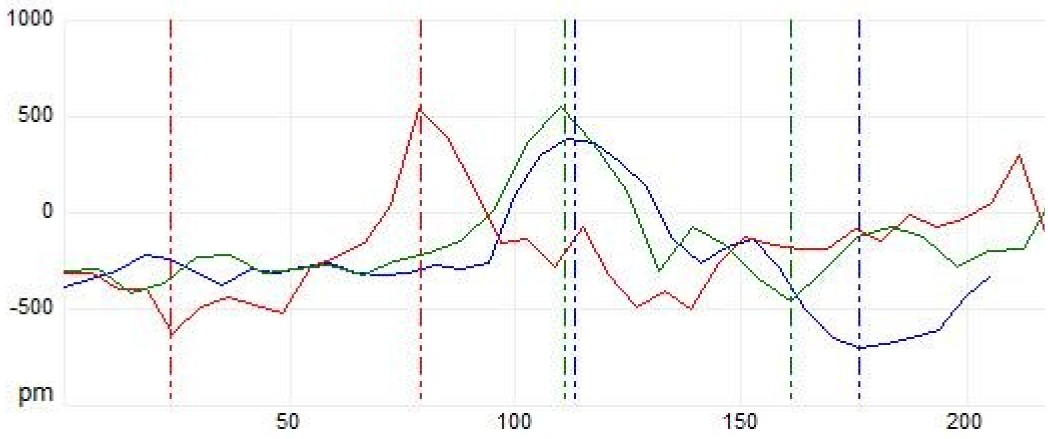

The heights measured are the following: Blue: 1.1 nm; Red: 1.2 nm and green: 1.0 nm. They were calculated as the difference between vertical sizes (same colors).

Figure S15. AFM experiments (tapping mode) of compound **2**.

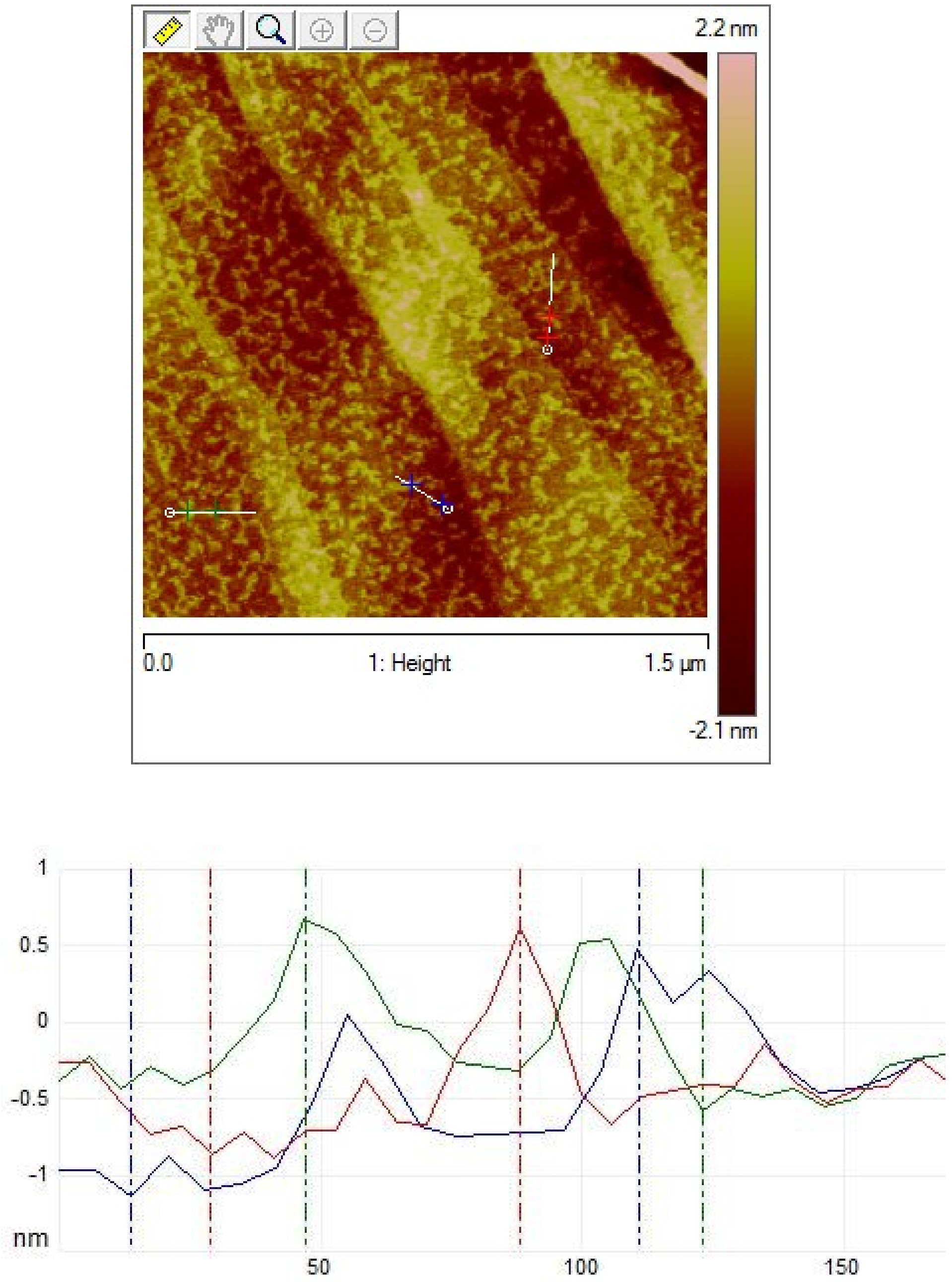

The heights measured are the following: Blue: 1.6 nm; Red: 1.5 nm and green: 1.2 nm. They were calculated as the difference between vertical sizes (same colors).

Figure S16. AFM experiments (contact mode – tapping mode) of compound **2**.

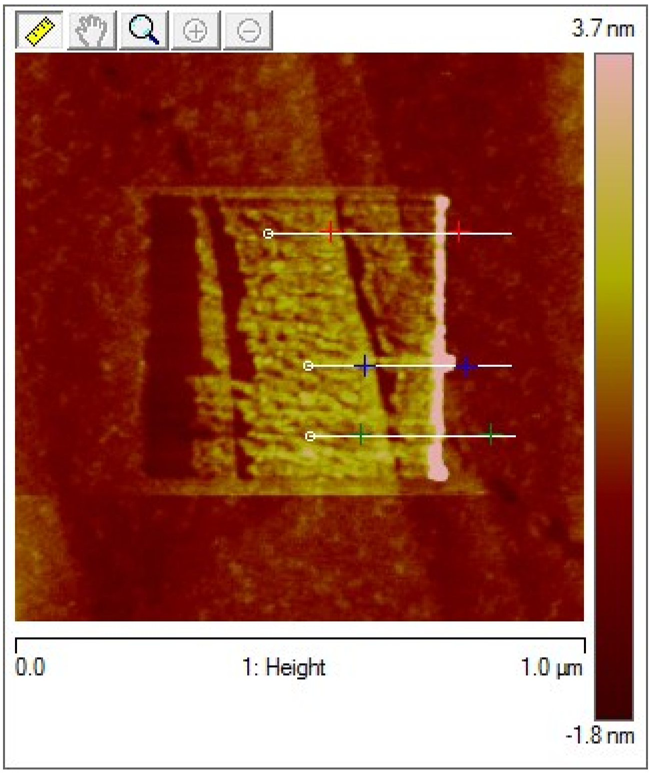

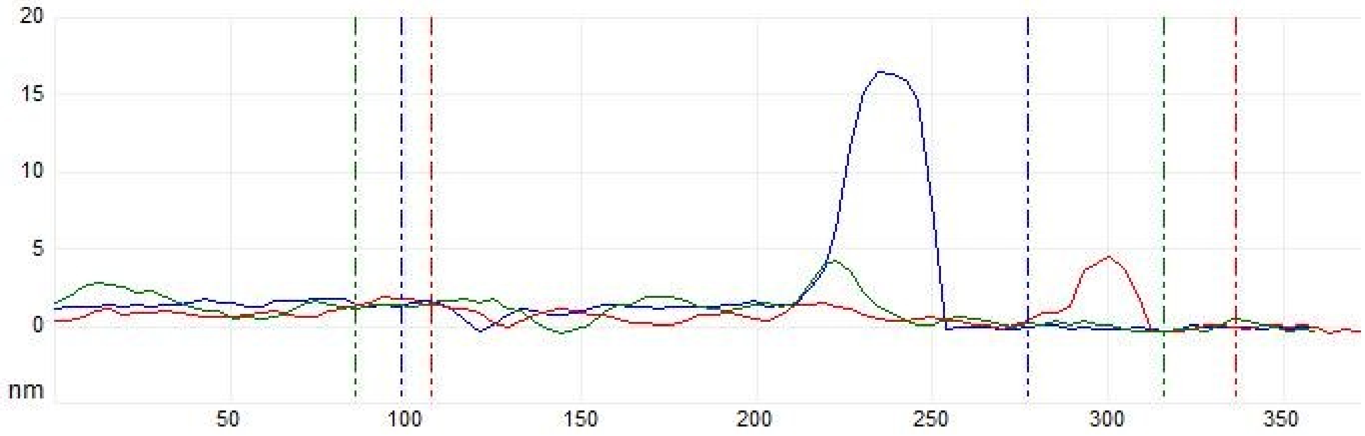

The heights measured are the following: Blue: 1.2 nm; Red: 1.3 nm and green: 1.4 nm. They were calculated as the difference between vertical sizes (same colors).

Figure S17. XPS experiments of a film of **2** spin-coated on HOPG.

The position of the spin-orbit splitting lines as well as the shape of the corresponding satellites are compatible with a formal Co^{II} electronic configuration [S1].

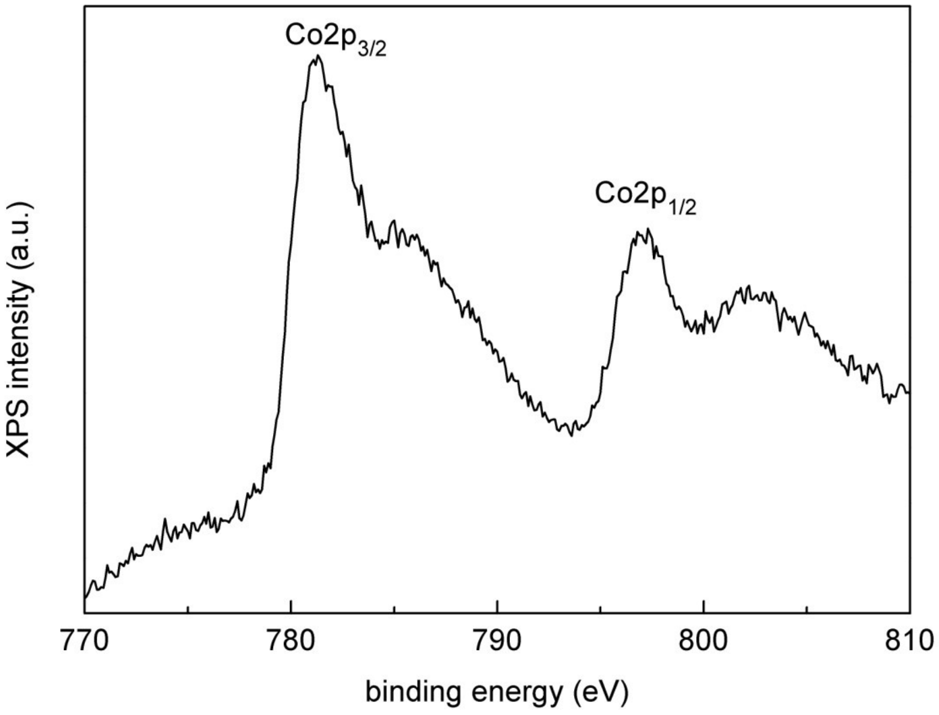

[S1] M. C. Biesinger, B. P. Payne, A. P. Grosvenor, L. W. M. Lau, A. R. Gerson, R. St. C. Smart, *Resolving surface chemical states in XPS analysis of first row transition metals, oxides and hydroxides: Cr, Mn, Fe, Co and Ni. Appl. Surf. Sci.* **2011**, *257*, 2717.

Table S1. Crystal data and structure refinement for compounds **1-2**

	**Compound (1)**	**Compound (2)**
**Formula**	$C_{80}H_{56}CoN_2O_4$	$C_{80}H_{54}CoN_2O_4$,$CHCl_3$
**M_r**	1168.20	1285.55
**Crystal system**	monoclinic	monoclinic
**Space group**	P21/c	P21/n
**λ (Å)**	0.77490	0.77490
**a (Å)**	11.4437(11)	17.249(3)
**b (Å)**	8.9623(9)	16.900(3)
**c (Å)**	29.228(3)	22.440(4)
**α (°)**	90.00	90,00
**β (°)**	94.705(2)	108.612(3)
**ϒ (°)**	90.00	90.00
**V ($Å^3$)**	2987.6(5)	6200(2)
**T [K]**	100(2)	100(2)
**Z**	2	4
**ρ (g/cm^3)**	1.229	1.377
**Shape and color**	plate & yellow	plate & orange
**Crystal size (mm^3)**	0.12x0.05x0.01	0.07x0.04x0.02
**Reflections**	4277	13457
**Parameters**	394	820
**Restraints**	0	0
**R_{int}**	0.0817	0.1171
**R_1[a]**	0.0468	0.0744
**wR2[b]**	0.1185	0.1627
**S**	0.987	0.943

[a] $R_1 = \sum| |F_o| - |F_c| |/\sum|F_o|$, para I>2σ(I). [b] $wR_2 = \{\sum[w(Fo^2 - F_c^2)^2]/\sum[w(F_o^2)^2]\}^{1/2}$, for all data.

[Co(9Accm)$_2$(py)$_2$] (1)

**Table S1. Selected interatomic distances [Å] and angles [°] for compound 1**			
**Co1 O1**	2.002(2)	**O2 Co1 O2**	180.00(8)
**Co1 O1**	2.002(2)	**O1 Co1 N1**	90.06(12)
**Co1 O2**	2.033(2)	**O1 Co1 N1**	89.94(12)
**Co1 O2**	2.033(2)	**O2 Co1 N1**	88.51(11)
**Co1 N1**	2.209(4)	**O2 Co1 N1**	91.49(11)
**Co1 N1**	2.209(4)	**O1 Co1 N1**	89.94(12)
**O1 Co1 O1**	180.00(11)	**O1 Co1 N1**	90.06(12)
**O1 Co1 O2**	90.13(9)	**O2 Co1 N1**	91.49(11)
**O1 Co1 O2**	89.88(9)	**O2 Co1 N1**	88.51(11)
**O1 Co1 O2**	89.88(9)	**N1 Co1 N1**	180.00(16)
**O1 Co1 O2**	90.12(9)	**O1 Co1 O2**	90.12(9)

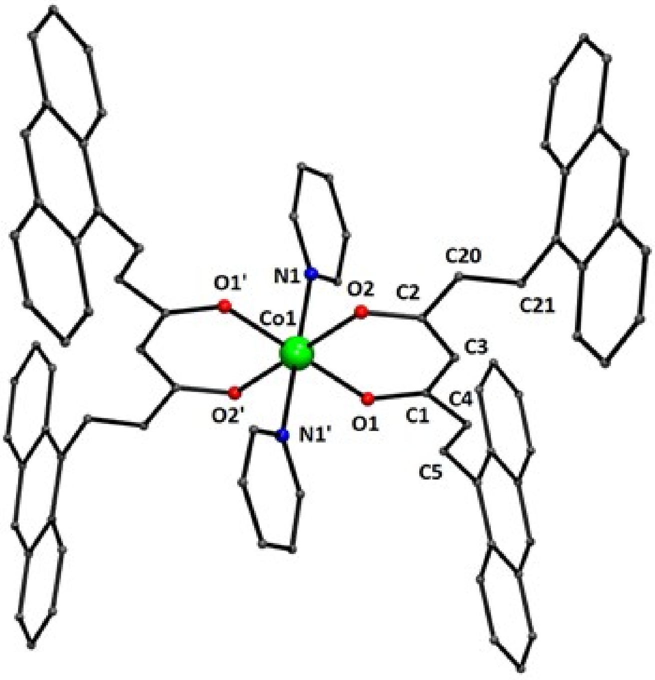

[Co(9Accm)$_2$(2,2'-bpy)] (2)

Table S1. Selected interatomic distances [Å] and angles [°] for compound 2

**Co1 O2**	2.012(3)	**O3 Co1 O1**	87.87(10)
**Co1 O4**	2.065(3)	**O2 Co1 N1**	167.39(12)
**Co1 O3**	2.069(3)	**O4 Co1 N1**	88.45(12)
**Co1 O1**	2.071(3)	**O3 Co1 N1**	101.17(12)
**Co1 N1**	2.109(4)	**O1 Co1 N1**	95.55(12)
**Co1 N2**	2.115(3)	**O2 Co1 N2**	91.16(12)
**O2 Co1 O4**	88.95(11)	**O4 Co1 N2**	88.48(11)
**O2 Co1 O3**	90.97(11)	**O3 Co1 N2**	174.37(11)
**O4 Co1 O3**	86.35(10)	**O1 Co1 N2**	97.41(11)
**O2 Co1 O1**	88.22(11)	**N1 Co1 N2**	76.44(14)
**O4 Co1 O1**	173.52(11)	**O3 Co1 O1**	87.87(10)

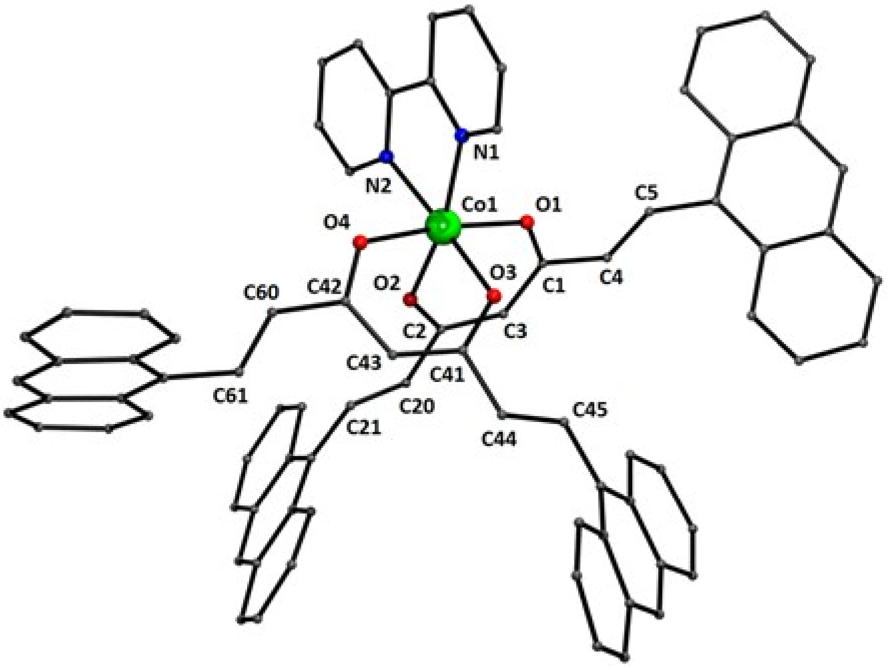

Table S2. ^{1}H NMR peaks of compounds **1**, **3** and **4**.

(1) *[Co(9Accm)$_2$(py)$_2$]*	*(3)* *[Co(9Accm)$_2$(3,5-(CH$_3$)$_2$-py)$_2$]*	*(4)* *[Co(9Accm)$_2$(dmf)$_2$]*	*Ref[1]:* *[Co(acac)$_2$(py)$_2$]*	*Ref[2]:* *[Co(acac)$_2$(3-(CH$_3$)-py)$_2$]*	
63.0	60.6	60.9			***-CH-***
38.0	28.8		58.7	44.7	***ortho-***
		18.2	3.1		**DMF**
13.2				18.9	***meta-***
11.5	10.8		28.5		***para-***
10.6	10.4	10.3			**9Accm**
9.4	8.9				
8.2	8.3	8.5			**9Accm**
7.4	7.6	7.4			**9Accm**
7.2	7.2	7.2			$CDCl_3$
		7.0			
	3.5				**methyl**
		2.8			
1.6	1.6	1.6			H_2O
		-4.0			**DMF**

[1] J. Chinese Chem. Soc, 23, 185-194 (1978)

[2] The Journal of Chemical Physics, 39, 1211 (1963)

Table S3. ^{1}H NMR peaks of compounds **2**, **5** and **6**.

(2) Co(9Accm)$_2$(2,2'-bpy)	*(5) Co(9Accm)$_2$(4,4'-(CH$_3$)$_2$-2,2'-bpy)*	*(6) Co(9Accm)$_2$(5,5'-(CH$_3$)$_2$-2,2'-bpy)*	*Ref[1]: Co(acac)$_2$(2,2'-bpy)]*	*Ref[2] Co(2,2'-bpy)$_3$Cl$_2$*	
89.6	86.3	84.8	74.5	81.0	***ortho-***
63.6	61.3	63.9	62.6	74.5	***meta'-***
47.5	46.9	46.5			***-CH-***
34.9	32.6		24.6	38.5	***meta-***
26.0	26.3	26.5			
13.1	13.1	13.1			**9Accm**
12.8		11.4	4.5	6.1	***para-***
9.5					
8.7	8.7	8.7			**9Accm**
8.5	8.5	8.5			**9Accm**
8.3	8,3	8,3			**9Accm**
7.7	7.7	7.7			**9Accm**
7.2	7.2	7.2			$CDCl_3$
		4.9			**methyl**
2.8	2.8				
1.6	1.6	1.6			H_2O
	-6.3				**methyl**
-22.4	-21.2	-21.2			**9Accm**

[1] J. Chinese Chem. Soc, 23, 185-194 (1978)

[2] Journal of the American Chemical Society, 90:8, April 10, 1968

Table S4. Comparison of magnetic data A', C and n published and this work.

	Ref 1	Ref. 2	Ref 3	Ref 4	This work
A'($s^{-1}K^{-1}$)	0.12 (1*, 0.05 T)<br>0.265 (2*, 0.05 T)<br>9.04 (3*, 0.18 T)<br>6.00 (4*, 0.08 T)<br>122.5 (5*, 0.25 T)	4.2 (#, 0.06 T)<br>7.2 (#, 0.17 T)<br>47.1 (#, 0.30 T)	-	-	447 (1*, 0.15 0T)<br>6688 (2*, 0.075 T)
C $s^{-1}K^{-n}$	$3{\cdot}10^{-3}$ (1*)<br>$5{\cdot}10^{-3}$ (2*)<br>$1.1{\cdot}10^{-2}$ (3*)<br>$4.7{\cdot}10^{-2}$ (4*)<br>$1.52{\cdot}10^{-1}$ (5*)	$5{\cdot}10^{-4}$	$4.66{\cdot}10^{-4}$	-	1.8 (1*)<br>5.3 (2*)
n	5 (1*)<br>5 (2*)<br>5 (3*)<br>4 (4*)<br>5 (5*)	5	8.6	8.7(1*)<br>4.9(2*)<br>11.2(3*)	5 (1*)<br>7.5(2*)

* Different compounds assigned with different numbers # Same compound measured at different magnetic fields

Ref 1 - J. M. Zadrozny, M. Atanasov, A. M. Bryan, C.-Y. Lin, B. D. Rekken, P. P. Power, F. Neese, J. R. Long, *Chem. Sci.* **2013**, 4, 125-138.

Ref 2 - S. Gómez-Coca, A. Urtizberea, E. Cremades, P. J. Alonso, A. Camon, E. Ruiz, F. Luis, *Nature Commun.* **2014**, 5, 4300 (1-8).

Ref 3 - F. Habib, I. Korobkov, M. Murugesu, *Dalton Trans.* **2015**, 44, 6368-6373.

Ref 4 - X.-C. Huang, C. Zhou, D. Shao, X.-Y. Wang, *Inorg. Chem.* **2014**, 53, 12671-12673.
